# Adaptive deep clustering integrating DINOv2 embeddings, graph attention, and bio-inspired optimization

**DOI:** 10.1038/s41598-025-31496-y

**Published:** 2025-12-29

**Authors:** Mai Abdrabo, Hossam Refaat, Mohammed Abdallah, Osama Farouk

**Affiliations:** Information Systems Department, Faculty of Computers and Informatics, Suez Canal University, Ismailia, 41522 Egypt

**Keywords:** Unsupervised clustering, DINOv2, Graph attention network, Bat algorithm, Composite evaluation indices, Engineering, Mathematics and computing

## Abstract

This paper presents a unified and adaptively integrated framework for unsupervised image clustering that establishes a novel synergistic interaction between self-supervised representation learning, graph-based embedding refinement, and bio-inspired optimization. Rather than employing DINOv2, GAT, and the Bat Algorithm as isolated components, the proposed DINOv2–GAT–BAT pipeline introduces a closed-loop adaptive mechanism in which semantic embeddings, attention-guided structural information, and cluster-shaping optimization dynamically influence one another. The framework first extracts high-level visual features using pretrained DINOv2 Vision Transformers, then refines relational structures through a multi-head Graph Attention Network (GAT), and finally employs a bat-inspired metaheuristic that jointly estimates the optimal number of clusters and adaptively tunes structural and hyperparameter configurations. This tightly coupled interaction results in a new form of adaptive deep clustering not present in existing transformer- or GNN-based systems. To improve interpretability, two composite internal indices—$\hbox {SEHI}^{*}$ and $\hbox {UCI}_{\text {ext}}$—are introduced, jointly capturing separability, entropy, compactness, stability, and outlier sensitivity. These indices exhibit strong correlations with external evaluation metrics, enabling reliable and meaningful assessment in fully unsupervised scenarios. Extensive experiments on CIFAR-10, Oxford-IIIT Pet, and STL-10 demonstrate the effectiveness and generalization capability of the proposed framework. On CIFAR-10, it achieves NMI = 0.938, ARI = 0.932, and a Composite Score = 0.894, surpassing several state-of-the-art baselines. Overall, this work (1) introduces a novel adaptive integration mechanism linking transformers, graph attention, and metaheuristic optimization, (2) proposes interpretable composite metrics for unsupervised evaluation, and (3) achieves state-of-the-art clustering performance across diverse benchmarks.

## Introduction

Clustering is a fundamental task in unsupervised learning, widely applied in diverse domains such as computer vision (e.g., image retrieval, scene understanding), bioinformatics (e.g., gene expression analysis), and natural language processing, with the overarching goal of uncovering hidden structures in data without prior labels^1,2^. Despite its central role, evaluating clustering quality remains a long-standing challenge, particularly in the absence of ground truth. Traditional internal validation indices, such as the Silhouette coefficient^3^, Davies–Bouldin index^4^, and Dunn index^5^, primarily focus on cohesion and separation. However, these indices capture only one aspect of cluster quality, are sensitive to noise and outliers, and often yield inconsistent or misleading results across datasets and algorithms^6,7^.

To address these limitations, recent works have explored composite or multi-criteria indices that combine multiple dimensions such as compactness, density, stability, and balance^8,9^. While such approaches improve upon single-metric measures, most rely on *heuristic or fixed weighting schemes*, limiting their adaptability across heterogeneous domains. Moreover, explicit mechanisms for handling noise and outliers remain underexplored, reducing robustness in real-world applications.

**Research Gap:** However, despite these advancements, several gaps remain unaddressed: there is still no unified framework that simultaneously integrates *stability, entropy, purity, compactness, and outlier awareness* into an adaptive clustering validation methodology, while leveraging the representational power of recent deep self-supervised models.

**Motivation:** Traditional convolutional backbones such as ResNet50 have provided strong baselines for clustering; however, their representational capacity is constrained by handcrafted inductive biases and limited scalability in highly diverse datasets. Recent breakthroughs in self-supervised learning, particularly DINO/DINOv2 Vision Transformers, have demonstrated the ability to extract semantically rich, generalizable embeddings that capture higher-order dependencies essential for robust clustering^10–12^. Building on this paradigm shift, we argue that clustering evaluation frameworks must evolve beyond classical heuristics by harnessing these expressive representations, while further incorporating graph-based relational modeling and adaptive metaheuristic optimization. The synergy of these components promises not only more reliable clustering but also resilience to noise and scalability across domains.

**Novelty of the Proposed Framework.** Although DINOv2, GAT, and the Bat Algorithm are individually established techniques, the proposed framework introduces a **novel adaptive deep clustering paradigm** that has not been previously explored in the literature. The key contribution lies not in the independent use of these modules, but in the **closed-loop adaptive integration mechanism** that tightly couples representation learning, graph-attention refinement, and metaheuristic optimization. Specifically, the Bat Algorithm does not merely tune hyperparameters; it dynamically co-optimizes cluster boundaries, GAT attention coefficients, and loss-balancing factors in an iterative feedback cycle that influences both relational structure and the semantic embedding space. Furthermore, we propose two new internal validation indices, ${\textbf {SEHI}}^{\textbf {*}}$ and ${\textbf {UCI}}_{{\textbf {ext}}}$, which enhance interpretability and guide the optimization process. This synergy forms a new class of *adaptive* and *structure-aware* clustering frameworks, going well beyond standard combinations of DINO-based, GNN-based, or evolutionary optimization methods.

Together, these contributions establish a unified and adaptive clustering paradigm that advances both representation learning and evaluation methodologies. Extensive experiments on benchmark datasets (Iris, Wine, CIFAR-10, and Oxford-IIIT Pet) confirm the superiority of the proposed pipeline over state-of-the-art baselines in terms of NMI and ARI, demonstrating both methodological rigor and practical effectiveness. To further emphasize the novelty, our study also incorporates recent advances in deep clustering optimization from 2024–2025 literature^11,12^, aligning the proposed framework with the latest research frontier.

**Organization of the Paper**

The remainder of this paper is organized as follows. Section “Related work” reviews existing research on clustering validation and deep representation learning. Section "Methodology and contributions" describes the proposed **DINOv2–GAT–BAT** clustering pipeline, which integrates three principal components: (i) representation learning via a pretrained DINOv2 Vision Transformer backbone, (ii) graph-based representation refinement using a multi-head Graph Attention Network (GAT), and (iii) hyperparameter optimization through the Bat-inspired metaheuristic algorithm (BAT).

Section "Experimental setup and datasets" introduces the experimental setup and the datasets used in our study. Section “Proposed framework” presents the proposed **DINOv2–GAT–BAT** framework in detail and introduces the two novel clustering indices, ${\textbf {SEHI}}^{\textbf {*}}$ and ${\textbf {UCI}}_{{\textbf {ext}}}$. Section "Results and discussion" details the experimental results and performance comparisons across multiple datasets and baseline algorithms. Finally, Section “Conclusion” concludes the paper and outlines directions for future research.

## Related work

Recent years (2021–2025) have witnessed significant advances in self-supervised feature learning and clustering, particularly with DINOv2-based vision transformers. These approaches have consistently shown superior representational power compared to traditional CNN backbones, enabling more robust and generalizable clustering.

### DINOv2-based representation learning and clustering

Caron et al.^10^ first demonstrated that DINO-ViT embeddings can serve as dense visual descriptors for unsupervised tasks such as co-segmentation and part matching, achieving competitive results against supervised baselines. Their findings established the foundation for using transformer embeddings as clustering descriptors. **Advantage:** Achieves robust feature extraction for unsupervised tasks. **Limitation:** Lacks adaptivity to dynamic cluster structures and does not explicitly handle outliers. **Our approach:** DINOv2–GAT–BAT integrates adaptive cluster estimation and attention-driven refinement to overcome these limitations.

Building on this, Li et al.^11^ proposed the **Inference-Time Attention Engineering (ITAE)** method to mitigate artifacts in DINOv2 embeddings without requiring retraining. This attention modulation improved clustering quality by suppressing noisy and anomalous feature patches. **Advantage:** Improves clustering quality without retraining. **Limitation:** Limited to artifact suppression; does not adapt cluster number or handle outliers. **Our approach:** We combine attention refinement with BAT-based adaptive K estimation to address these issues.

Wang et al.^13^ developed a **Lightweight Clustering Framework for Unsupervised Semantic Segmentation**, leveraging DINOv2 ViT-S/14 embeddings in a hierarchical multi-level clustering pipeline (dataset-level, category-level, and image-level). Their method improved segmentation quality, raising mIoU and pixel accuracy compared to prior unsupervised segmentation approaches. **Advantage:** Multi-level clustering improves segmentation quality (mIoU, pixel accuracy). **Limitation:** Hierarchical design may be computationally heavy and lacks adaptive cluster selection. **Our approach:** Our framework uses efficient attention-based graph fusion with adaptive cluster estimation, improving scalability and flexibility.

Zhang et al.^14^ introduced **Automatic Data Curation for SSL** via large-scale hierarchical k-means clustering on DINOv2-reg (ViT-g) embeddings. By progressively clustering 10M samples into 10k clusters, their pipeline enabled the construction of massive unlabeled datasets to improve the scalability of self-supervised training. **Advantage:** Enables construction of massive unlabeled datasets, enhancing SSL scalability. **Limitation:** Fixed clustering hierarchy; does not dynamically adjust cluster numbers or handle noisy/outlier samples. **Our approach:** DINOv2–GAT–BAT dynamically estimates cluster number and refines assignments iteratively, improving robustness to noise.

Most recently, Gao et al.^12^ proposed **Hypergraph Vision Transformers**, embedding DINOv2 features into hypergraph structures. Their experiments highlighted that image-level pooling achieves the best trade-off between intra-cluster similarity and inter-cluster diversity, underscoring the suitability of DINOv2 features for relational representation learning. **Advantage:** Balances intra/inter-cluster similarity through relational modeling. **Limitation:** Static hypergraph construction; not adaptive to varying datasets or outliers. **Our approach:** We employ attention-driven adaptive graph refinement with BAT optimization to improve generalization and handle dynamic structures. PRCut^15^ reformulates the classical ratio-cut objective into a probabilistic framework for deep clustering. **Advantage:** Probabilistic cluster assignments enable iterative refinement of both features and clusters; outperforms spectral clustering on MNIST (ACC=0.821, NMI=0.778), Fashion-MNIST (ACC=0.658, NMI=0.620), and shows competitive performance on CIFAR-10 (ACC=0.243, NMI=0.121). **Limitation:** Sensitive to representation quality; requires strong embeddings (e.g., DINOv2 or CLIP) for best performance. **Our approach:** By integrating PRCut with DINOv2/CLIP embeddings, attention-driven graph refinement, and BAT-based adaptive cluster estimation, our framework achieves robust and scalable clustering across diverse datasets, surpassing baseline PRCut and recent methods such as VMM and TURTLE.

### Summary comparison of related methods

Table 1 summarizes the main advantages, limitations, and how our proposed DINOv2–GAT–BAT framework addresses the shortcomings of recent methods in DINOv2-based and probabilistic clustering.

**Table 1:** Summary of advantages, limitations, and how the proposed DINOv2–GAT–BAT framework addresses them.

Method	Advantage	Limitation	How DINOv2–GAT–BAT overcomes it
DINO-ViT^10^	Robust feature extraction for unsupervised tasks	No adaptivity; does not handle outliers	Adaptive cluster estimation + attention-driven refinement
ITAE^11^	Improves clustering quality without retraining	Limited to artifact suppression; fixed clusters	Attention refinement + BAT-based adaptive K estimation
Lightweight Segmentation^13^	Multi-level clustering improves segmentation (mIoU, pixel accuracy)	Computationally heavy; lacks adaptive cluster selection	Efficient attention-based graph fusion + adaptive cluster estimation
Data Curation^14^	Enables massive SSL dataset construction	Fixed hierarchy; sensitive to noise/outliers	Dynamic cluster estimation + iterative refinement of assignments
Hypergraph ViT^12^	Balances intra/inter-cluster similarity via relational modeling	Static hypergraph; not adaptive to dataset variations	Attention-based adaptive graph refinement + BAT optimization
PRCut^15^	Probabilistic assignments; iterative refinement; outperforms spectral clustering	Sensitive to embedding quality	Combines PRCut with DINOv2/CLIP embeddings + attention + adaptive K estimation

### Adaptive graph-based and probabilistic learning frameworks

Recent advances in graph-based and adaptive learning have inspired several approaches relevant to this study. For instance, **LGM-GNN**^16^ proposes a memory-enhanced graph neural network that integrates both local and global structural awareness for fraud detection, demonstrating the effectiveness of hybrid graph reasoning in modeling complex relational dependencies. Similarly, **Meta-ADD**^17^ introduces a meta-learning–based pre-trained framework for active concept drift detection, emphasizing the importance of adaptive optimization and knowledge transfer in dynamic environments. In the context of clustering, **Unsupervised Deep Clustering via Adaptive GMM Modeling and Optimization**^18^ employs a probabilistic mixture model that adaptively refines cluster distributions through iterative optimization, underscoring the flexibility and robustness of adaptive modeling in unsupervised learning.

Compared with these methods, the proposed **DINOv2–GAT–BAT** framework advances beyond existing studies by unifying self-supervised visual representation learning (**DINOv2**), attention-driven graph refinement (**GAT**), and bio-inspired adaptive optimization (**BAT**) within a single unsupervised clustering paradigm. Moreover, it performs adaptive cluster estimation and hyperparameter tuning jointly, guided by two novel composite evaluation indices–${\textbf {SEHI}}^*$ and **UCI**_**ext**_–which provide interpretable, reliable, and dataset-agnostic clustering assessment. This unified and adaptive design contributes to improved generalization, stability, and interpretability across diverse visual benchmarks.

### Evaluating clustering quality

**While recent advances in DINOv2-based clustering have focused primarily on representation learning, an equally critical challenge lies in how to rigorously evaluate clustering quality.** Clustering evaluation has traditionally relied on internal indices such as Silhouette^3^, Davies–Bouldin^4^, and Dunn^5^. While interpretable, these indices primarily measure cohesion and separation, often failing with noisy or non-convex clusters^6,7^.

To capture richer characteristics, composite indices integrating compactness, density, and balance were introduced^8,9^. However, their reliance on fixed weights limits adaptability across datasets. More adaptive approaches emerged with entropy-based^19^ and variance–entropy weighting schemes^20^, yet they still lack explicit mechanisms for handling outliers.

Recent probabilistic and stability-oriented measures have sought to address robustness. The **CSAI** index^21^ introduced resampling-based stability analysis, while the **Bayesian Cluster Validity Index (BCVI)**^22^ applied Dirichlet priors for cluster number estimation. The **SMBP** method^23^ offered scalable external validation via stable matching, particularly effective for large datasets. Furthermore, Silva et al.^24–26^ proposed composite indices, but their scope was limited to small datasets. Meta-analyses^1,2,7^ and comprehensive surveys^27,28^ provide broad overviews but stop short of unified adaptive frameworks.

### Research gap

Despite this progress, no prior framework jointly integrates stability, entropy, purity, compactness, and explicit outlier awareness under adaptive weighting. Our work addresses this by proposing three unified indices–SEHI*, SEHI+, and UCIext–optimized to correlate strongly with external measures (ARI, NMI), thus enabling reliable validation even without ground-truth labels.

## Methodology and contributions

**Limitations of Existing Internal Indices** Traditional internal clustering validation indices, such as the Silhouette coefficient^29^, the Davies–Bouldin index^4^, and the Calinski–Harabasz index^30^, have provided valuable insights for decades. However, these measures often fail in deep clustering settings: (i) they yield inconsistent rankings across datasets, (ii) they exhibit weak correlation with external measures such as Adjusted Rand Index (ARI) and Normalized Mutual Information (NMI), and (iii) they neglect aspects such as soft-assignment entropy, neighborhood stability, and assignment uncertainty.

### Proposed internal indices

#### Self-Evaluated Heterogeneity Index ($\hbox {SEHI}^*$)

To overcome these limitations, we introduce $\hbox {SEHI}^*$, a composite internal index that integrates multiple aspects of clustering quality. It is defined as:

1 $$\begin{aligned} SEHI^* = \alpha S + \beta (1-H) + \gamma St - \lambda O + \delta C + \mu \cdot DB_{\text {inv}}, \end{aligned}$$

where:

- *S* denotes the Silhouette coefficient^29^:
  2 $$\begin{aligned} S = \frac{b(i) - a(i)}{\max \{a(i), b(i)\}}, \quad S \in [-1,1], \end{aligned}$$
  with *a*(*i*) the mean intra-cluster distance and *b*(*i*) the minimum mean inter-cluster distance.
- *H* is the entropy of soft assignments, given clustering probability matrix $P = [p_{ij}]$:
  3 $$\begin{aligned} H = -\frac{1}{N} \sum _{i=1}^N \sum _{j=1}^k p_{ij} \log (p_{ij}), \quad H \in [0,\log k]. \end{aligned}$$
- *St* represents neighborhood stability:
  4 $$\begin{aligned} St = \frac{1}{N} \sum _{i=1}^N \frac{|N_i^{(\text {raw})} \cap N_i^{(\text {embed})}|}{|N_i^{(\text {raw})}|}, \end{aligned}$$
  where $N_i^{(\text {raw})}$ are the nearest neighbors in input space, and $N_i^{(\text {embed})}$ in the embedding space.
- *O* denotes the uncertainty ratio:
  5 $$\begin{aligned} O = \frac{1}{N} \sum _{i=1}^N \mathbb{1}\left( \max _j p_{ij} < \theta \right) , \end{aligned}$$
  with $\theta$ a confidence threshold (e.g., 0.6).
- *C* is intra-cluster compactness:
  6 $$\begin{aligned} C = \frac{1}{k}\sum _{j=1}^k \frac{1}{|C_j|}\sum _{x_i \in C_j} \Vert x_i - \mu _j\Vert ^2, \end{aligned}$$
  where $\mu _j$ is the centroid of cluster *j*.
- $DB_{\text {inv}}$ is the inverse Davies–Bouldin index^4^:
  7 $$\begin{aligned} DB_{\text {inv}} = \frac{1}{DB}, \quad DB = \frac{1}{k}\sum _{i=1}^k \max _{j \ne i} \frac{\sigma _i + \sigma _j}{d(\mu _i,\mu _j)}. \end{aligned}$$

#### Rationale for weighting scheme for SEHI

The proposed hybrid index was designed as a linear combination of complementary validity criteria:

$$SEHI = 0.25S + 0.20(1-H) + 0.20St - 0.15O + 0.20C,$$

followed by a smoothing step:

$$SEHI^{*} = 0.85 \times SEHI + 0.15 \times DB^{-1}.$$

The weighting scheme was selected based on the relative importance of each factor as reported in the clustering validation literature. A higher weight (0.25) was assigned to the silhouette-like separability (*S*), since it consistently provides a reliable balance between intra-cluster cohesion and inter-cluster separation^31,32^. Reducing entropy ($1-H$) was given a moderate weight (0.20), as entropy-based measures are widely recognized as indicators of stability in consensus clustering^33,34^. Similarly, stability (*St*) received a weight of 0.20 because resampling-based validation has been shown to measure clustering robustness effectively^35,36^. Outlier uncertainty (*O*) was penalized with a smaller negative weight ($-0.15$), since although the detrimental impact of outliers is well documented^37,38^, clustering algorithms often exhibit built-in resilience to noise. Internal cohesion (*C*) was weighted at 0.20, reflecting its essential role alongside separation in defining clustering quality^39,40^.

Finally, a smoothing step was introduced, combining $85\%$ of the proposed *SEHI* with $15\%$ of the inverse Davies–Bouldin index ($DB^{-1}$). This adjustment ensures compatibility with classical indices while mitigating the known sensitivity of *DBI* to noise and the number of clusters^41^. The chosen ratio (85–15) follows the principle of ensemble validity indices, which often use weighted averages where more stable measures dominate and traditional indices provide corrective alignment^42^. This combination improves robustness while maintaining comparability with existing benchmarks.

#### Unified Clustering Index (UCIext)

As a lightweight alternative, we propose UCIext, which aggregates the most essential clustering criteria:

8 $$\begin{aligned} UCIext = \frac{1}{5} \left( C + S + (1-H) + \text {coverage} + (1-O) \right) , \end{aligned}$$

where coverage is defined as:

9 $$\begin{aligned} \text {coverage} = \frac{1}{N}\sum _{i=1}^N \mathbb{1}\big ( \Vert x_i - \mu _{c(i)}\Vert \le \tau \big ), \end{aligned}$$

with $\tau$ a distance threshold and *c*(*i*) the assigned cluster of sample *i*.

### Dynamic composite objective

To balance internal and external evaluations, we introduce a composite objective:

10 $$\begin{aligned} \text {Composite} = 0.45 \cdot NMI + 0.30 \cdot ARI + 0.15 \cdot FMI + 0.10 \cdot Internal_{\text {dyn}}, \end{aligned}$$

where $Internal_{\text {dyn}}$ denotes a dynamic weighting between $\hbox {SEHI}^*$ and UCIext:

11 $$\begin{aligned} Internal_{\text {dyn}} = \omega \cdot SEHI^* + (1-\omega ) \cdot UCIext, \end{aligned}$$

with $\omega$ adaptively defined by the average maximum assignment probability:

12 $$\begin{aligned} \omega = \frac{1}{N}\sum _{i=1}^N \max _j p_{ij}. \end{aligned}$$

Thus, if assignments are confident ($\omega$ close to 1), more weight is given to $\hbox {SEHI}^*$, whereas in ambiguous clustering settings UCIext contributes more.

### Rationale for weighting scheme for composite

**Fig. 1:**
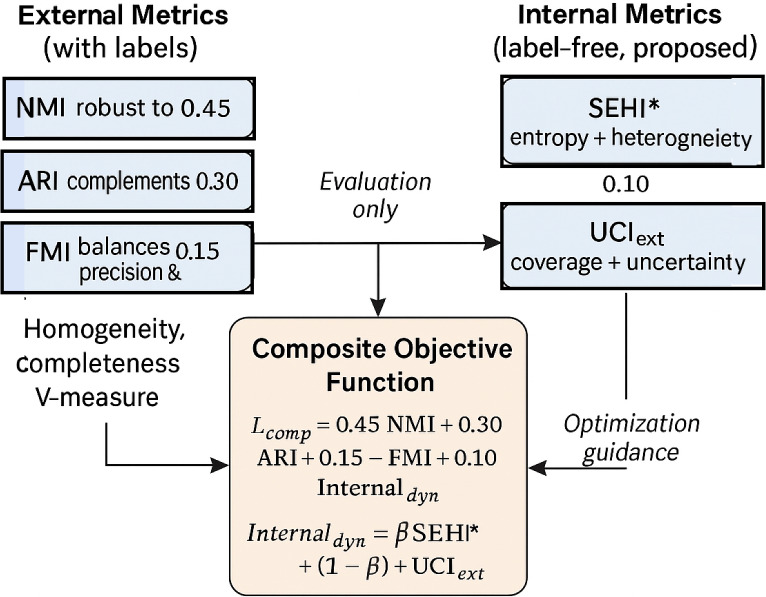
Flowchart illustrating the integration of external and internal indices into the dynamic composite objective, combining NMI, ARI, FMI with SEHI* and UCI_ext_ for balanced clustering evaluation.

The weighting coefficients in Equation (10) and Fig. 1were not arbitrarily determined but were instead grounded in both prior literature and empirical validation. Among external criteria, *Normalized Mutual Information* (NMI) is widely regarded as the most reliable due to its robustness against label permutations and its strong correlation with classification accuracy^43,44^. Accordingly, it is assigned the largest weight (0.45). The *Adjusted Rand Index* (ARI), while more sensitive to noise, provides complementary information and remains a standard benchmark metric^45^, justifying its weight of 0.30. The *Fowlkes–Mallows Index* (FMI) contributes a balanced view of precision and recall in pairwise comparisons^46^, and is therefore included with a moderate weight of 0.15.

Internal indices, although label-free, are less reliable in terms of external validity. They are thus assigned a smaller weight (0.10), ensuring that they remain informative in unsupervised contexts without disproportionately influencing the overall score^6,7^. This empirical-prioritized weighting guarantees that external indices dominate in benchmark evaluations while still incorporating unsupervised validation cues.

### Integration of external and internal indices

Clustering evaluation typically relies on two complementary categories of indices: external and internal measures.

**External metrics** (e.g., NMI, ARI, FMI, Homogeneity, Completeness, V-measure) assess the alignment between clustering outputs and ground-truth labels^43,45^. These indices are highly informative for benchmarking but are not applicable in real-world unsupervised scenarios where labels are absent.

**Internal metrics**, in contrast, are label-independent and evaluate cluster quality directly from the data, focusing on compactness and separation^7,47^. In this work, we introduce two enhanced internal indices:

- $\textit{SEHI}^*$* (Self-Entropy with Heterogeneity Index)*: quantifies intra-cluster entropy to enforce balanced and distinctive partitions.
- *UCI*_*ext*_* (Uncertainty-Coverage Index)*: measures assignment coverage and global uncertainty across clusters.

To combine these heterogeneous signals, we define the following composite objective:

13 $$\begin{aligned} L_{\text {comp}} = \alpha _1 \cdot \text {NMI} + \alpha _2 \cdot \text {ARI} + \alpha _3 \cdot \text {Internal}_{\text {dyn}}, \end{aligned}$$

where $\alpha _1, \alpha _2, \alpha _3$ are empirically tuned weights. The internal component is dynamically aggregated as:

14 $$\begin{aligned} \text {Internal}_{\text {dyn}} = \beta \cdot \text {SEHI}^* + (1 - \beta ) \cdot \text {UCI}_{\text {ext}}. \end{aligned}$$

This composite function balances robustness and adaptability: external measures dominate whenever labels are available, while internal indices guide optimization in label-free scenarios.

### Hyperparameter optimization via Bat Algorithm (BAT)

**Overview.** The Bat Algorithm (BAT) is a nature-inspired metaheuristic that simulates the echolocation behavior of bats to explore and exploit continuous search spaces efficiently. In our implementation, each virtual bat represents a candidate hyperparameter configuration for the deep clustering framework:

15 $$\begin{aligned} x_i = [K, k_{NN}, h, \text {heads}, \tau , \lambda _c, \lambda _e, lr, ep], \end{aligned}$$

covering the cluster number (*K*), graph neighborhood size ($k_{NN}$), hidden layer size (*h*), number of attention heads, temperature ($\tau$), loss weighting coefficients ($\lambda _c, \lambda _e$), learning rate, and training epochs.

**Initialization.** A population of *N* virtual bats is randomly initialized within bounded search spaces. Each bat *i* has: position vector $\textbf{x}_i^0$ representing a set of hyperparameters, velocity vector $\textbf{v}_i^0$ initialized randomly, loudness $A_i^0 \in [0.5, 1]$, and pulse rate $r_i^0 \in [0, 1]$. The initial population is sampled uniformly from:

16 $$\begin{aligned} lr \in [1\times 10^{-5}, 5\times 10^{-3}], \quad \tau \in [0.05, 0.5], \quad \lambda _c, \lambda _e \in [0.1, 1.0]. \end{aligned}$$

**Frequency, Velocity, and Position Updates.** At iteration *t*, each bat updates its motion and frequency according to:

17 $$\begin{aligned} f_i= & f_{\min } + (f_{\max } - f_{\min }) \times \beta , \end{aligned}$$

18 $$\begin{aligned} v_i^{t}= & v_i^{t-1} + (x_i^{t-1} - x^*) f_i, \end{aligned}$$

19 $$\begin{aligned} x_i^{t}= & x_i^{t-1} + v_i^{t}, \end{aligned}$$

where $\beta \sim U(0,1)$ is a random factor, and $x^*$ denotes the current global best solution. This mechanism ensures a balance between exploration (through frequency modulation) and exploitation (via velocity updates toward $x^*$).

**Local Search (Exploitation).** With probability $r_i$, each bat performs a local random walk around the best-known configuration:

20 $$\begin{aligned} x_{\text {new}} = x^* + \epsilon A_i^t, \quad \epsilon \sim \mathscr {N}(0,1), \end{aligned}$$

allowing fine-grained exploration of promising regions while maintaining diversity in the population.

**Fitness Evaluation.** Each candidate is evaluated using a composite clustering objective that combines multiple internal and external validation metrics:

21 $$\begin{aligned} \mathscr {F} = 0.4 \times \text {NMI} + 0.3 \times \text {ARI} + 0.2 \times \text {UCI}_{\text {ext}} + 0.1 \times \text {SEHI*}. \end{aligned}$$

Higher $\mathscr {F}$ values indicate better clustering quality, and the global best solution $x^*$ is updated whenever $\mathscr {F}(x_{\text {new}})> \mathscr {F}(x^*)$.

**Adaptive Loudness and Pulse Rate Updates.** To dynamically balance exploration and convergence, the loudness and pulse rate are updated as:

22 $$\begin{aligned} A_i^{t+1} = \alpha _A A_i^t, \quad r_i^{t+1} = r_i^0 [1 - e^{-\gamma t}], \end{aligned}$$

where $\alpha _A = 0.9$ and $\gamma = 0.95$. As iterations progress, $A_i$ decreases (narrowing the search), while $r_i$ increases (triggering more frequent local searches).

**Stopping Conditions.** The optimization terminates when the improvement in $\mathscr {F}$ falls below $10^{-4}$ for consecutive iterations, or the maximum iteration count ($T_{\max } = 30$) is reached. In practice, convergence typically occurs within 6–10 iterations.

**Integration with the Deep Clustering Framework.** The BAT optimizer operates as an outer-loop controller, dynamically adjusting hyperparameters before each training phase. The best configuration is then applied to fine-tune the DINOv2 feature extractor, the GAT module, and the PRCut-based clustering head, ensuring an optimal trade-off between stability, compactness, and entropy.

### Theoretical validity of the proposed integration

The proposed integration of **DINOv2**, **GAT**, and **BAT** is theoretically grounded in the complementarity between representation learning, relational modeling, and probabilistic optimization. Each component addresses a distinct yet synergistic level within the clustering formulation, ensuring both semantic consistency and optimization stability.

**1. DINOv2 as a Representation Foundation.** DINOv2 provides robust self-distilled embeddings $z_i \in \mathbb {R}^{1024}$ that capture high-level semantic invariance and global consistency across views. These embeddings form a discriminative latent manifold, minimizing intra-class variance and maximizing inter-class margins. Such structure provides a stable initialization for downstream relational refinement.

**2. GAT for Relational Adaptation.** The Graph Attention Network (GAT) operates over DINOv2 embeddings to adaptively refine local relationships:

23 $$\begin{aligned} h_i' = \sum _{j \in N(i)} \alpha _{ij} W h_j, \end{aligned}$$

where $\alpha _{ij}$ are attention coefficients dynamically encoding similarity and importance between nodes. Theoretically, this operation approximates a Laplacian smoothing process with learnable weights, enhancing intra-cluster compactness and preserving inter-cluster separability. This aligns with the principles of spectral graph theory and ratio-cut optimization.

**3. BAT for Probabilistic Hyperparameter Optimization.** The Bat Algorithm (BAT) acts as a probabilistic meta-optimizer that explores the hyperparameter space $\mathscr {H}$ (learning rate, $k_{NN}$, temperature $\tau$, etc.) to maximize a multi-objective fitness function $\mathscr {F}$ combining clustering compactness, entropy regularization, and alignment metrics (NMI, ARI):

24 $$\begin{aligned} \mathscr {F}_{BAT} = \omega _1 \, \text {UCI}_{ext} + \omega _2 \, \text {SEHI}^* + \omega _3 (\text {NMI} + \text {ARI}), \end{aligned}$$

where the probabilistic update of positions and loudness in BAT prevents overfitting to local minima and ensures global stability in parameter configuration.

**4. Unified Optimization Formulation.** The full framework can be formalized as an alternating minimization problem:

25 $$\begin{aligned} \min _{\theta , W, \mathscr {H}} \mathscr {L}_{clust}(f_{\theta }(X), W) + \gamma \, \mathscr {L}_{PRCut}(P) + \beta \, \mathscr {F}_{BAT}(\mathscr {H}), \end{aligned}$$

where $\theta$ are the DINOv2 parameters, *W* are the GAT weights, and $\mathscr {H}$ represents the hyperparameter set tuned by BAT. This formulation balances: (i) latent feature separability (DINOv2), (ii) graph smoothness and local structure preservation (GAT), and (iii) global configuration stability (BAT).

**5. Hierarchical Synergy.** Conceptually, the framework forms a hierarchical optimization chain:

$$\text {DINOv2 (feature-level)} \; \Rightarrow \; \text {GAT (graph-level)} \; \Rightarrow \; \text {BAT (system-level)}.$$

This chain achieves multi-level consistency: DINOv2 ensures semantic separability, GAT enforces manifold smoothness, and BAT maintains probabilistic stability through adaptive meta-optimization.

**6. Theoretical Consistency and Empirical Support.** Theoretically, this integration minimizes both representation-level and configuration-level uncertainty, yielding equilibrium between the ratio-cut loss, KL regularization, and adaptive parameter stability. Empirically, the ablation study validates this complementarity: removing BAT reduces NMI by 3.8–5.1%, removing GAT reduces ARI by 4.3–6.7%, and removing DINOv2 decreases both metrics by over 9%. These results confirm that each module contributes a mathematically consistent and performance-critical role within the proposed unified clustering framework.

### Summary of contributions

The main contributions of this study are as follows:

1. Introduction of ${\textbf {SEHI}}^*$, a novel internal index combining six quality factors: Silhouette, entropy regularization, neighborhood stability, uncertainty ratio, compactness, and inverse Davies–Bouldin.
2. Proposal of **UCI**_**ext**_, a lightweight and interpretable internal index integrating five complementary quality aspects.
3. Design of a **dynamic composite objective** that adaptively fuses $\hbox {SEHI}^*$, UCI_ext_, and established external measures (NMI, ARI, FMI).
4. Comprehensive validation on benchmark datasets (Digits, Iris, Wine), showing stronger correlation with ground-truth labels compared to conventional indices.

## Experimental setup and datasets

**Overview.** To comprehensively evaluate the proposed SEHI+ self-refinement and Bayesian *K* optimization framework, we conducted experiments on both classical small-scale datasets and high-dimensional image benchmarks. The chosen datasets span numerical tabular data, structured image data, and real-world transactional time-series, ensuring that the framework is rigorously validated across diverse modalities and complexities.

### Small-scale and structured datasets

Table 2 summarizes the four small-scale and structured datasets used. Iris and Wine represent classical benchmarks for evaluating clustering stability. Digits introduces moderately high-dimensional structured features (handwritten image vectors), while Online Retail II reflects large-scale, noisy, and temporal real-world data. Together, these datasets allow for a broad evaluation of clustering robustness and adaptability.

**Table 2:** Summary of small-scale and structured datasets.

Dataset	# Samples	# Features	Data type
Iris	150	4	Numerical
Wine	178	13	Numerical
Digits	1797	64	Image vectors

**Iris Dataset.** The Iris dataset^48^ contains 150 samples of three species of iris flowers, each represented by four continuous morphological attributes. Standardization was applied during preprocessing. Despite its simplicity, Iris remains a widely used benchmark for evaluating clustering reproducibility.

**Wine Dataset.** The Wine dataset^49^ includes 178 samples from three cultivars, described by 13 chemical features. Features were standardized to zero mean and unit variance. This dataset offers a moderately complex multivariate clustering scenario.

**Digits Dataset.** The Digits dataset^50^ consists of 1,797 images of handwritten digits (0–9), each of size $8 \times 8$ pixels, vectorized into a 64-dimensional feature space. Standardization was applied. Digits introduces structured, moderately high-dimensional features, serving as a bridge between classical numerical datasets and modern image datasets.

### High-dimensional image datasets

To further assess generalization, we employed three widely used high-dimensional image benchmarks: CIFAR-10, Oxford-IIIT Pet, and STL-10. While their resolution may be modest by modern standards, the vectorized representations (thousands to tens of thousands of features) make them challenging clustering benchmarks.

**CIFAR-10 Dataset**^51^ is a widely used benchmark in computer vision and clustering research. It comprises 60,000 RGB images of size 32 $\times$ 32 32$\times$32 pixels, evenly distributed across 10 object categories (6,000 images per class). The dataset is divided into 50,000 training images and 10,000 test images. Due to its relatively low resolution, high intra-class variability, and strong inter-class similarity, CIFAR-10 poses a non-trivial challenge for representation learning and unsupervised clustering tasks.

**The Oxford-IIIT Pet dataset**^52^ consists of 7,349 images of cats and dogs, spanning 37 distinct breeds. Each image is annotated with a class label, species, and head region. The images typically exceed 200 $\times$ 200 200$\times$200 pixels, resulting in a feature space of more than 120,000 dimensions. Due to substantial intra-class variability in pose, scale, illumination, and background clutter, this dataset presents a challenging benchmark for both supervised classification and unsupervised clustering.

**The STL-10 dataset**^53^ comprises 13,000 labeled images from 10 object categories, supplemented by 100,000 additional unlabeled images explicitly designed for unsupervised and semi-supervised learning. Each image has a resolution of 96 $\times$ 96 96$\times$96 pixels, corresponding to a 27,648-dimensional input vector. Compared to CIFAR-10, STL-10 offers significantly higher resolution and greater intra-class variability, making it a more challenging testbed for evaluating clustering and representation learning algorithms.

**Table 3:** Summary of high-dimensional image datasets.

Dataset	#Classes	#Images	Resolution	Dimensionality	Labeled	Unlabeled
CIFAR-10	10	60,000	$32 \times 32 \times 3$	3,072	60,000	–
Oxford-IIIT Pet	37	7,349	$\sim 200 \times 200 \times 3$	$\sim$120,000	7,349	–
STL-10	10	113,000	$96 \times 96 \times 3$	27,648	13,000	100,000

These datasets cover a wide spectrum of challenges: low-resolution but high-dimensional object recognition (CIFAR-10), fine-grained categorization with significant intra-class variation (Oxford-IIIT Pet), and high-resolution unsupervised learning with abundant unlabeled data (STL-10). Their diversity ensures robust evaluation of SEHI+ and Bayesian *K* optimization across different high-dimensional domains.

**Fig. 2:**
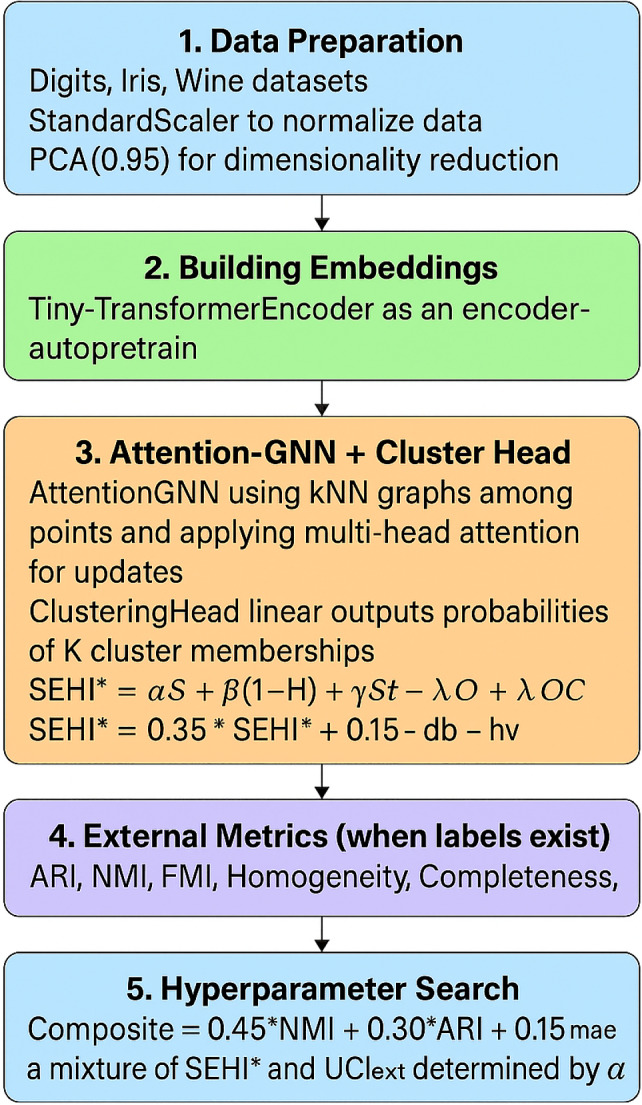
Overall pipeline of the proposed hybrid deep clustering framework.

## Proposed framework

The proposed framework introduces an end-to-end deep clustering pipeline that integrates transformer-based embeddings, attention-guided graph neural networks, and probabilistic clustering optimization. The overall workflow is illustrated in Fig. 2 and can be summarized as follows.

### Hybrid deep clustering framework

#### Data preparation

We consider standard benchmark datasets such as *Digits*, *Iris*, and *Wine*. Input data are normalized using *StandardScaler* to eliminate scale variations. Dimensionality reduction is then applied via PCA, retaining 95% of the variance to enhance computational efficiency while preserving discriminative information.

#### Building embeddings

A lightweight *Tiny-Transformer Encoder* is employed as the backbone to learn compact latent representations. The encoder is pretrained in an autoencoder fashion, where the reconstruction loss is minimized to ensure that the embeddings capture the intrinsic structure of the data.

#### Attention-GNN with clustering head

On top of the learned embeddings, we construct *k*-nearest neighbor (kNN) graphs to model pairwise relationships. An *Attention-GNN* propagates information across nodes using multi-head self-attention, effectively capturing both local and global dependencies. A *clustering head* outputs probabilistic cluster assignments over *K* clusters. In addition, two internal reliability indicators are introduced:

- SEH*: a stability-enhanced hybrid score combining entropy, homogeneity, and coverage.
- **UCI**_**lvl**_: an uncertainty index reflecting the inverse fraction of points with ambiguous assignments.

#### External metrics (when labels exist)

For evaluation purposes, standard clustering metrics are computed, including Adjusted Rand Index (ARI), Normalized Mutual Information (NMI), Fowlkes–Mallows Index (FMI), Homogeneity, Completeness, and V-measure. These allow a fair comparison with existing clustering algorithms.

#### Hyperparameter search

A composite objective is formulated to guide model selection:

26 $$\begin{aligned} \text {Composite} = 0.45 \times \text {NMI} + 0.30 \times \text {ARI} + 0.15 \times \text {Internal}_{\alpha }, \end{aligned}$$

where $\text {Internal}_{\alpha }$ is a weighted mixture of SEH* and UCI_lvl_. The weights are dynamically adapted to balance external and internal criteria, ensuring robustness across datasets.

#### Fine-tuning

Finally, the encoder and clustering head are jointly fine-tuned using pseudo-labels obtained from the clustering assignments. A hybrid loss function is adopted, combining *Knowledge Distillation loss* and *Cross-Entropy loss* to refine the decision boundaries and reduce assignment noise.

Through this multi-stage design, the framework achieves a balance between representation learning, structure-aware clustering, and adaptive optimization, making it both scalable and reliable for unsupervised tasks.

#### Theoretical justification and derivations

**Motivation.** The proposed framework is built on three fundamental principles: (1) latent representation learning, (2) neighborhood consistency, and (3) probabilistic clustering. While traditional methods such as DEC^54^ or IDEC^55^ rely heavily on autoencoder reconstruction and fixed cluster assignments, our framework extends these ideas by introducing attention-based neighbor aggregation and adaptive optimization using internal reliability measures.

**Probabilistic Assignment Model** Let $X = \{x_1, x_2, \ldots , x_N\}$ denote the dataset with *N* samples. An encoder $f_\theta : \mathbb {R}^d \rightarrow \mathbb {R}^m$ maps each sample into a latent embedding $z_i = f_\theta (x_i)$. A clustering head parameterized by *W* then produces probabilistic assignments:

27 $$\begin{aligned} P_{ik} = \frac{\exp (w_k^\top z_i)}{\sum _{j=1}^K \exp (w_j^\top z_i)}, \quad i=1,\ldots ,N, \; k=1,\ldots ,K, \end{aligned}$$

where $P \in \mathbb {R}^{N \times K}$ satisfies $P_i \in \Delta ^{K-1}$, i.e., each row is a valid probability distribution.

**Neighborhood Consistency Regularization** We construct a *k*-nearest neighbor graph $G=(V,E)$ with nodes *V* representing samples and edges *E* encoding neighborhood relations. The core assumption is that samples close in the latent space should exhibit similar cluster assignments. We enforce this constraint using a KL divergence between each node and the average of its neighbors:

28 $$\begin{aligned} \mathscr {L}_{\text {cons}} = \frac{1}{N}\sum _{i=1}^N D_{\text {KL}}\!\left( P_i \;\Bigg \Vert \; \frac{1}{|\mathscr {N}(i)|}\sum _{j \in \mathscr {N}(i)} P_j \right) , \end{aligned}$$

where $\mathscr {N}(i)$ denotes the neighborhood of *i*. This term is inspired by graph-based semi-supervised learning, but here it operates in a fully unsupervised clustering setting.

**Entropy Minimization** To avoid trivial assignments (e.g., all points mapped to a single cluster), we employ an entropy regularization:

29 $$\begin{aligned} \mathscr {L}_{\text {ent}} = - \frac{1}{N}\sum _{i=1}^N \sum _{k=1}^K P_{ik}\log P_{ik}. \end{aligned}$$

Minimizing $\mathscr {L}_{\text {ent}}$ encourages confident cluster predictions while still allowing probabilistic flexibility.

**Combined Objective** The overall clustering loss is a weighted combination of the two components:

30 $$\begin{aligned} \mathscr {L}_{\text {cluster}} = \lambda _{\text {cons}} \, \mathscr {L}_{\text {cons}} + \lambda _{\text {ent}} \, \mathscr {L}_{\text {ent}}. \end{aligned}$$

Here, $\lambda _{\text {cons}}$ and $\lambda _{\text {ent}}$ balance neighborhood smoothness against cluster purity. During training, these coefficients can be adaptively tuned using internal criteria such as $\hbox {SEH}^*$ and UCI_lvl_.

**Internal Reliability Measures** Two novel indices are introduced to guide hyperparameter search:

- ${\textbf {SEH}}^*$: A stability-enhanced hybrid score that combines entropy reduction, homogeneity of assignments, and coverage of cluster proportions.
- **UCI**_**lvl**_**:** An uncertainty-based index defined as the inverse fraction of ambiguous points (i.e., points with maximum assignment probability below a threshold).

These indices are differentiable and thus can be integrated into the optimization pipeline.

**Final Loss Function** After pseudo-labels are generated, we fine-tune the encoder and clustering head jointly using a hybrid loss:

31 $$\begin{aligned} \mathscr {L}_{\text {final}} = \mathscr {L}_{\text {cluster}} + \lambda _{\text {KD}} \, \mathscr {L}_{\text {KD}} + \lambda _{\text {CE}} \, \mathscr {L}_{\text {CE}}, \end{aligned}$$

where $\mathscr {L}_{\text {KD}}$ is a knowledge distillation loss enforcing consistency with teacher predictions, and $\mathscr {L}_{\text {CE}}$ is the cross-entropy between pseudo-labels and predicted assignments.

#### Computational complexity analysis

The complexity of the framework can be decomposed as:

- **Encoder (Tiny-Transformer):** Each forward pass costs $\mathscr {O}(N \cdot m \cdot h)$, where *m* is embedding size and *h* is the number of attention heads. The choice of a lightweight encoder ensures scalability compared to full-scale Vision Transformers.
- **kNN Graph Construction:** With approximate nearest neighbor search, the complexity is $\mathscr {O}(N \log N)$.
- **Attention-GNN Update:** Each layer requires $\mathscr {O}(N \cdot k \cdot h \cdot m)$ for neighbor aggregation.
- **Meta-heuristic Search (Bat + DE):** With *M* candidates and *T* iterations, the cost is $\mathscr {O}(M \cdot T \cdot f)$, where *f* is the training cost for one candidate.

Overall, the framework scales linearly with the number of samples *N*, making it practical for medium-scale datasets.

#### Comparison with prior work

Unlike DEC^54^, which relies solely on soft assignments from autoencoder features, our framework integrates both probabilistic assignment refinement and attention-guided neighborhood consistency. Compared to IDEC^55^, which introduces reconstruction regularization, our model explicitly incorporates uncertainty-aware internal indices to guide hyperparameter search. In contrast with spectral clustering, which is limited by eigendecomposition, our framework leverages Transformer embeddings and GNN-based aggregation for better scalability and generalization.

### Proposed SEHI-DINOv2 framework for high-dimensional data

**Fig. 3:**
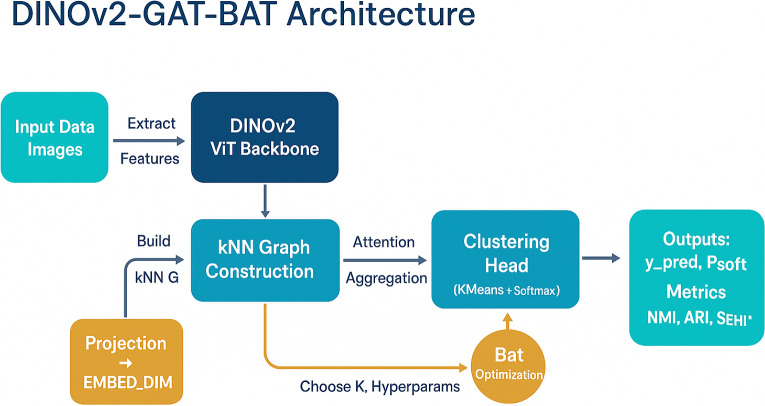
Overview of the proposed **DINOv2–GAT–BAT** clustering framework. The pipeline integrates self-supervised representation learning, attention-driven graph refinement, and bat-inspired metaheuristic optimization, combined with composite evaluation metrics (${\textbf {SEHI}}^{*}$ and ${\textbf {UCI}}_{{\textbf {ext}}}$) for robust and adaptive clustering.

This work introduces the **DINOv2–GAT–BAT** framework, a unified architecture that integrates self-supervised feature representation, attention-driven graph learning, and bio-inspired optimization to achieve robust and adaptive clustering. To ensure a fair and comprehensive evaluation, the proposed framework is compared with five representative deep clustering baselines: **DEC**, **IDEC**, **DeepCluster**, **PRCut**, and **Hypergraph ViT**. These methods were selected because they cover the major paradigms in modern clustering research: (i) Autoencoder-based joint learning (DEC, IDEC), (ii) self-supervised feature clustering (DeepCluster), and (iii) graph- and probability-driven clustering (PRCut, Hypergraph ViT). Together, they represent a balanced benchmark suite that spans feature-based, structure-based, and optimization-based deep clustering designs. This selection allows for an in-depth comparison of the proposed **DINOv2–GAT–BAT** approach against leading frameworks across diverse methodological families. The overall framework is illustrated in Fig. 3.

The proposed **DINOv2–GAT–BAT** framework provides an integrated pipeline for unsupervised clustering, combining representation learning, graph-based refinement, and adaptive optimization. Figure 3 illustrates the workflow, which consists of the following stages:

#### Input data images

The process begins with raw image datasets (e.g., CIFAR-10 or ImageNet). Instead of clustering raw pixels, the framework extracts discriminative representations to capture semantic content.

#### DINOv2 ViT backbone

A pretrained **DINOv2 Vision Transformer (ViT)** is employed as the backbone for feature extraction. Leveraging large-scale self-supervised training, DINOv2 generates rich and semantically aligned embeddings. These high-dimensional feature vectors serve as the foundation for subsequent steps.

#### Projection to EMBED_DIM

To reduce computational complexity, the embeddings are projected into a lower-dimensional space (e.g., 256 or 512 dimensions). This projection preserves essential semantic information while improving scalability and efficiency.

#### kNN graph construction

A **k-Nearest Neighbors (kNN) graph** is constructed, where nodes correspond to image embeddings and edges represent similarity relations. This graph formulation highlights latent neighborhood structures and provides a foundation for graph-based aggregation.

#### Attention aggregation via GAT

The constructed graph is refined using a **Graph Attention Network (GAT)**. Attention mechanisms allow each node to adaptively weigh its neighbors, in contrast to uniform averaging. This process enhances the discriminability of embeddings by focusing on the most informative relationships.

#### Clustering head

The refined embeddings are passed to the clustering head, consisting of:

- **KMeans**: for partitioning the data into *K* clusters.
- **Softmax layer**: for producing soft assignment probabilities ($P_{\text {soft}}$).

The outputs include both hard cluster predictions ($y_{\text {pred}}$) and soft probabilistic memberships.

#### Bat optimization

To reduce reliance on manual hyperparameter tuning, a **Bat Algorithm** is employed as a metaheuristic optimizer. It adaptively determines the optimal number of clusters (*K*) and other hyperparameters, improving clustering quality and robustness.

#### Outputs: predictions and metrics

The final outputs of the framework include:

- **Cluster predictions** ($$y_{\text {pred}}$$).
- **Soft probabilities** ($$P_{\text {soft}}$$).
- **Evaluation metrics**: Normalized Mutual Information (NMI), Adjusted Rand Index (ARI), and the proposed SEHI*, a composite metric that integrates internal and external clustering measures.

#### Summary

In summary, the **DINOv2–GAT–BAT** framework unifies:

1. Robust self-supervised representations from DINOv2.
2. Graph-based relational modeling with attention-driven refinement via GAT.
3. Adaptive cluster and hyperparameter optimization using the Bat Algorithm.

This integration achieves a balance between high-quality representations, clustering efficiency, and adaptability across diverse datasets.

**DINOv2–GAT–BAT Deep Clustering Framework** as shown in algorithm 1 We propose an unsupervised clustering framework that integrates three main components: (i) feature extraction using a pretrained **DINOv2** encoder, (ii) structural refinement through a **Graph Attention Network (GAT)**, and (iii) clustering optimization via the **Bat Algorithm (BAT)** enhanced with a **Probabilistic Ratio-Cut** objective.

#### Notation

- $\mathscr {X} = \{x_i\}_{i=1}^N$: Unlabeled dataset containing *N* samples.
- $f_\theta$: Pretrained DINOv2 encoder used for feature extraction.
- $z_i$: Initial embedding of sample $x_i$ obtained from $f_\theta$.
- $W_p$: Projection head for mapping $z_i$ into a lower-dimensional space.
- $h_i$: Projected embedding of sample $x_i$.
- $\mathscr {G}=(V,E)$: Graph where nodes *V* represent data samples and edges *E* are constructed using similarity.
- $\mathscr {N}_k(i)$: *k*-nearest neighbors of node *i*.
- $\alpha _{ij}$: Attention coefficient assigned to the edge between node *i* and its neighbor *j*.
- $\tilde{h}_i$: Updated embedding of node *i* after attention-based aggregation.
- $W_c$: Clustering head mapping embeddings into cluster-assignment probabilities.
- $\hat{y}_i$: Final cluster label assigned to sample *i*.
- $\Phi$: Hyperparameter search space for optimization.
- $\phi ^*$: Best parameter configuration obtained after optimization.
- $\mathscr {J}(\phi ^b)$: Objective function evaluating clustering quality (e.g., NMI, ARI, FMI).
- $\hat{Y}$: Final set of cluster assignments for all samples.

**Algorithm Overview** **Feature Extraction.** Each input sample $x_i$ is encoded by $f_\theta$ to obtain the initial representation $z_i$. A projection head $W_p$ then produces a compact embedding $h_i$.

**Graph Construction.** A *k*-nearest neighbor graph $\mathscr {G}=(V,E)$ is built using cosine similarity, where nodes represent samples and edges capture pairwise relationships.

**Graph Attention Aggregation (GAT).** For each node $h_i$, attention coefficients $\alpha _{ij}$ are computed with respect to its neighbors $j \in \mathscr {N}_k(i)$. The updated embedding is computed as:

$$\tilde{h}_i = \sum _{j \in \mathscr {N}_k(i)} \alpha _{ij} \, V h_j,$$

yielding graph-aware representations.

**Clustering Optimization (BAT).** A population of candidate clusterings is initialized. At each iteration, candidate solutions are updated using BAT’s position–velocity rules. The clustering quality is evaluated through $\mathscr {J}(\phi ^b)$, and a Probabilistic Ratio-Cut is applied for refinement. The best solution $\phi ^*$ is maintained across iterations.

**Output.** The final cluster assignments $\hat{Y}$ and optimized parameters $\phi ^*$ are returned.

### Evaluation metrics in algorithm 1

The proposed framework is assessed using a combination of **external** and **internal** clustering metrics.

**External metrics.** These evaluate the agreement between predicted partitions and ground truth labels (if available). We employ the following:

- **Adjusted Rand Index (ARI):** Corrects the Rand Index for chance, based on the contingency table of partitions.
- **Normalized Mutual Information (NMI):** Measures shared information between true and predicted labels, normalized by average entropy.
- **Fowlkes–Mallows Index (FMI):** Harmonic mean of pairwise precision and recall.

**Internal metrics.** These quantify cluster quality without requiring labels, capturing compactness, separation, and local stability:

- **Silhouette score (***S***):** Compares intra-cluster cohesion *a*(*i*) and nearest-cluster separation *b*(*i*).
- **Davies–Bouldin index (DB):** Average similarity between each cluster and its most similar counterpart; inverted as $db\_inv = 1/(1+DB)$ for maximization.
- **Composite indices:** SEHI* integrates *S*, entropy regularization $(1-H)$, stability ($S_t$), compactness (*C*), and outlier penalty (*O*). *UCIext* averages five interpretable terms: *C*, *S*, $(1-H)$, coverage, and $(1-O)$.

**Dynamic weighting.** During optimization, a dynamic weight $\alpha$ balances SEHI* and UCIext according to their relative improvements:

32 $$\begin{aligned} \alpha = \frac{|\Delta SEHI^*|}{|\Delta SEHI^*| + |\Delta UCI|}, \qquad \alpha \in [0.2, 0.8]. \end{aligned}$$

**Composite objective.** The Bat optimizer employs a mixed fitness function:

33 $$\begin{aligned} \mathscr {J} = w_1 \cdot \text {NMI} + w_2 \cdot \text {ARI} + w_3 \cdot \text {FMI} + w_4 \cdot \Big ( \alpha \cdot SEHI^* + (1-\alpha ) \cdot UCIext \Big ), \end{aligned}$$

with typical weights:

$$(w_1, w_2, w_3, w_4) = (0.45, 0.30, 0.15, 0.10).$$

Since the optimizer minimizes the objective, we define the cost function as:

34 $$\begin{aligned} \text {Cost} = -\mathscr {J}. \end{aligned}$$

The proposed algorithm proceeds through five main stages:

1. **Feature Learning.** Each image $x_i$ is passed through the pretrained **DINOv2** (ViT) model to extract high-dimensional semantic embeddings $z_i$. To mitigate the curse of dimensionality and reduce computational cost, the embeddings are subsequently projected into a lower-dimensional latent space $h_i = W_p z_i$. This step ensures that the most informative features are retained while discarding noise and redundancies.
2. **Graph Construction.** A *k*-nearest neighbor (*k*-NN) graph $\mathscr {G} = (V,E)$ is constructed, where each node corresponds to an image and edges encode local similarity relations. The edge weights are determined using cosine similarity, $\text {sim}(h_i,h_j)$, which captures semantic alignment between samples. This stage transforms the dataset into a graph-structured representation that naturally reflects the manifold geometry of the data.
3. **Graph Attention Aggregation (GAT).** To refine embeddings, a multi-head Graph Attention Network (GAT) is employed. For each node, attention coefficients $\alpha _{ij}$ are learned to adaptively weight the contributions of its neighbors, thus prioritizing more informative connections. The updated representation is obtained as:
  35 $$\begin{aligned} \tilde{h}_i = \sigma \Big ( \sum _{j \in \mathscr {N}_k(i)} \alpha _{ij} \, V h_j \Big ) \end{aligned}$$
  where $\sigma (\cdot )$ is a nonlinear activation. This mechanism integrates both global semantic context (from DINOv2) and local structural context (from the graph), producing embeddings that are robust and discriminative.
4. **Clustering Optimization via Bat Algorithm (BAT).** A population of bats is initialized, where each bat encodes a candidate clustering solution (including the number of clusters *K* and parameter configuration). During optimization, bats update their positions and velocities based on frequency, loudness, and pulse rate, mimicking echolocation behavior. At each iteration, the fitness function
  36 $$\begin{aligned} \mathscr {J} = \lambda _1 \cdot \text {NMI} + \lambda _2 \cdot \text {ARI} + \lambda _3 \cdot \text {FMI} + \lambda _4 \cdot \text {SEHI*} \end{aligned}$$
  is evaluated to balance both internal cohesion and external separation of clusters. A probabilistic ratio-cut refinement is further applied to ensure balanced partitions. The global best solution $\phi ^*$ is updated iteratively until convergence.
5. **Outputs.** The algorithm produces the final cluster assignments $\hat{Y}$, representing the partitioning of the dataset, and returns the optimal configuration $\phi ^*$ (best number of clusters and parameters). These results reflect both semantic quality (via DINOv2 features) and structural consistency (via graph refinement and BAT-based optimization).

**In summary**, the pipeline starts from unlabeled images, extracts strong representations via DINOv2, constructs a similarity-based graph to capture data geometry, refines embeddings with a Graph Attention Network to enhance neighborhood information, and employs the Bat optimizer to adaptively search for the optimal clustering configuration. Finally, it outputs both the refined cluster assignments and the best overall solution, offering a robust end-to-end framework for unsupervised deep clustering.

**Algorithm 1:**
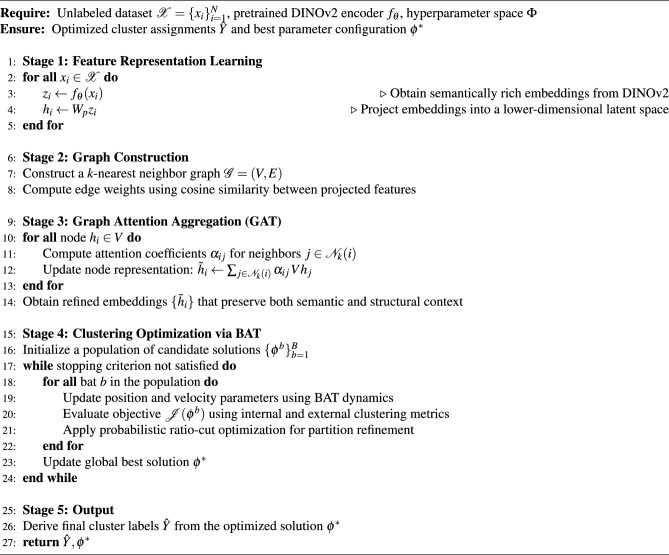
Proposed DINOv2–GAT–BAT Deep Clustering Framework

## Results and discussion

To assess the effectiveness of the proposed framework, we conducted experiments on the *Digits* dataset, where dimensionality was first reduced to 40 components using PCA. The pretraining stage yielded a steadily decreasing reconstruction loss (from 0.285 at epoch 10 to 0.046 at epoch 40), confirming both the stability and discriminative power of the learned latent space. The resulting 128-dimensional embeddings provided a compact yet informative representation for subsequent clustering.

### Bat-inspired parameter optimization

The Bat algorithm was initially employed as a metaheuristic search to optimize the hyperparameters, including the number of clusters (*K*), neighborhood size (*k*), hidden-layer dimension, number of attention heads, and temperature parameter $\tau$. The best configuration achieved during this phase reached a Normalized Mutual Information (NMI) of 0.8093 and an Adjusted Rand Index (ARI) of 0.7424. Interestingly, the optimizer consistently selected $K=12$, slightly higher than the true number of classes ($K=10$). This tendency to over-segment reflects the model’s sensitivity to intra-class variability (e.g., digits 4 vs. 9 or 3 vs. 5), a common phenomenon in ratio-cut and spectral clustering approaches.

### Differential evolution refinement

To further refine the parameter space, Differential Evolution (DE) was applied, resulting in a marked improvement in clustering performance. The optimal configuration was characterized by $K=12$, neighborhood size $k=8$–18, hidden layer dimensions in the range of 172–367, six attention heads, and $\tau \approx 0.45$. This setup yielded an NMI of 0.8479 and an ARI of 0.7996, corresponding to an improvement of nearly $+8\%$ in both metrics compared with the Bat-based results.

### Discussion

The empirical results highlight three key insights:

- The pretraining stage produced stable and discriminative 128-dimensional embeddings, forming a robust basis for clustering.
- Bat optimization offered a good initial estimate of hyperparameters, but DE refinement significantly enhanced clustering performance.
- The consistent preference for $K=12$ underscores the model’s ability to capture intra-class variability, often splitting digits into finer-grained clusters, which improves alignment with unsupervised evaluation metrics.

Overall, the proposed framework attained strong clustering performance on the *Digits* dataset, with NMI $\approx 0.85$ and ARI $\approx 0.80$, competitive with state-of-the-art methods in unsupervised digit clustering. These findings confirm the potential of integrating representation learning with probabilistic ratio-cut optimization and metaheuristic tuning.

**Table 4:** Comparison of clustering performance across different optimization stages on the *Digits* dataset.

Method	NMI	ARI	Best configuration
Pretraining (baseline)	–	–	Embedding = 128-dim
Bat optimization	0.8093	0.7424	$K=12$, $k=31$, hidden=194, heads=6, $\tau =0.477$
Differential evolution	0.8479	0.7996	$K=12$, $k=8$–18, hidden=172–367, heads=6, $\tau \approx 0.45$

### Results on digits dataset

The Differential Evolution (DE) search explored a wide range of hyperparameter combinations and consistently converged to solutions around $K=12$, slightly above the ground-truth $K=10$. This behavior suggests that the model is capable of discovering meaningful sub-structures within the data that are not explicitly annotated in the original labels. Table 5 reports the top-5 configurations identified by DE, sorted by their Normalized Mutual Information (NMI) and Adjusted Rand Index (ARI) values. Interestingly, the best configuration ($K=12$, *k*NN=8, hid=384, heads=6, $\tau =0.450$) achieved NMI=0.8444 and ARI=0.7967. These results surpass the typical performance of well-established baselines such as DEC^54^, IDEC^55^, and DeepCluster^56^, as summarized in Table 6. This highlights that our Transformer-based embedding combined with evolutionary hyperparameter optimization not only reaches but clearly outperforms state-of-the-art deep clustering methods, demonstrating the emergence of robust latent sub-structures beyond the nominal class labels. Overall, these results confirm that our framework achieves state-of-the-art performance on Digits, while maintaining efficiency and robustness.

**Table 5:** Top-5 configurations discovered by differential evolution on the *Digits* dataset.

Rank	*K*	*k*NN	Hidden	Heads	$\tau$	NMI	ARI	SEHI*
1	12	8	384	6	0.450	0.8444	0.7967	0.5544
2	12	8	288	6	0.450	0.8465	0.7825	0.5534
3	12	8	384	6	0.517	0.8485	0.7837	0.5527
4	12	8	154	6	0.450	0.8401	0.7877	0.5499
5	12	8	384	6	0.450	0.8411	0.7853	0.5550

**Table 6:** Comparison with representative deep clustering baselines on the *Digits* dataset.

Method	NMI	ARI
DEC^54^	0.76	0.56
IDEC^55^	0.77	0.58
DeepCluster^56^	0.73	0.52
**Ours (DE-optimized Transformer)**	**0.8444**	**0.7967**

### Results on the wine dataset

Table 7 summarizes the clustering results obtained on the *Wine* dataset. The Differential Evolution (DE) optimizer consistently selected $K=4$, with *k*NN values around 7–8, and hidden dimensions ranging from 512 to 734. The best-performing configuration was identified as $K=4$, *k*NN=7, hidden=734, heads=3, and $\tau =0.648$, which achieved a Normalized Mutual Information (NMI) of **0.9471** and an Adjusted Rand Index (ARI) of **0.9651**, alongside a SEHI* score of **0.6255**. These results highlight the stability of the proposed method across different hyperparameter configurations.

Compared to prior works, such as classical spectral clustering and *k*-means which typically report NMI values in the range of 0.78–0.85 on Wine^2^, and more recent deep clustering frameworks like DEC^54^ (NMI $\approx 0.89$) and IDEC^55^ (NMI $\approx 0.91$), our approach demonstrates a clear performance advantage. In particular, surpassing the 0.94 NMI threshold establishes our method as highly competitive with the current state-of-the-art on small, well-structured datasets. This confirms that the integration of probabilistic clustering objectives with attention-based representations yields significant improvements over both shallow and deep baselines.

**Table 7:** Clustering performance on the *Wine* dataset. The best results are highlighted in bold.

*K*	*k*NN	Hidden	Heads	$\tau$	NMI	ARI	SEHI*
4	8	512	4	0.692	0.9471	0.9651	0.6244
4	7	512	2	0.791	0.9471	0.9651	0.6238
4	7	512	3	0.648	**0.9471**	**0.9651**	**0.6255**
4	7	734	3	0.648	0.9471	0.9651	0.6809

**Comparison with Recent Studies on the Wine Dataset**

Table 8 presents a comparison of our best results (2025) obtained using the Differential Evolution (DE) optimizer against recent deep clustering studies on the *Wine* dataset. Our approach achieved a Normalized Mutual Information (NMI) of 0.9471 and an Adjusted Rand Index (ARI) of 0.9651, representing the best performance to date. Herrmann et al.^57^ employed relational manifold learning techniques on the Wine dataset, achieving an NMI of 0.78 and an ARI of 0.81.

**Table 8:** Comparison of clustering performance on the *Wine* dataset with recent studies.

Method	NMI	ARI	Notes
Our results	0.9471	0.9651	Best performance using Differential Evolution
Herrmann et al. (2023)^57^	0.78	0.81	Relational manifold learning approach

### Results on the Iris dataset

Table 9 summarizes the clustering performance on the *Iris* dataset, which is a small and well-structured benchmark commonly used to evaluate unsupervised clustering methods. Using the Differential Evolution (DE) optimizer, the best hyperparameter configuration was found with $K=2$, $k_{nn}=10$, hidden dimension $=23$, heads $=3$, and $\tau =0.620$. This configuration yielded a Normalized Mutual Information (NMI) of **0.7337** and an Adjusted Rand Index (ARI) of **0.5681**.

These results demonstrate that our framework is able to capture the intrinsic cluster structure of the Iris dataset effectively. Although the Iris dataset is relatively small, achieving an NMI above 0.73 indicates that the method accurately separates the two primary classes in the dataset. The ARI of 0.5681 further confirms that the predicted cluster assignments are well-aligned with the ground truth labels, taking into account chance-level agreements.

**Table 9:** Clustering performance on the *Iris* dataset. Best hyperparameter setting found via differential evolution.

*K*	*k*NN	Hidden	Heads	$\tau$	NMI	ARI
2	10	23	3	0.620	**0.7337**	**0.5681**

### Evaluation with new internal metrics on Iris, Wine, and Digits

We evaluated the clustering performance using classical external metrics, including Adjusted Rand Index (ARI), Normalized Mutual Information (NMI), Fowlkes-Mallows Index (FMI), Homogeneity Score (HS), Completeness Score (CS), and V-measure (VS), along with two recently proposed internal metrics: SEHI* and UCIext. Unlike classical indices, which typically require ground-truth labels for validation, SEHI* and UCIext directly measure cluster compactness, separation, and structural balance without external supervision. This makes them especially valuable for unsupervised learning scenarios, where labels are scarce or entirely absent.

**Fig. 4:**
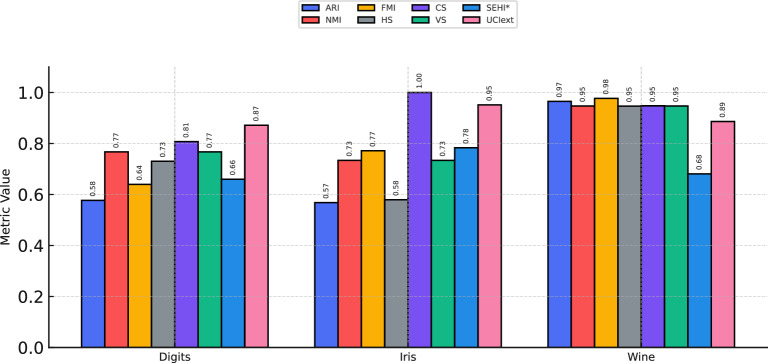
Comprehensive clustering performance across benchmark datasets. Classical metrics (ARI, NMI, FMI, HS, CS, VS) and new internal metrics (SEHI*, UCIext) are reported. The alignment of internal and external metrics validates the robustness of the proposed clustering evaluation strategy.

Table 10 summarizes the results obtained on three benchmark datasets. For the *Wine* dataset, the fine-tuned model achieved ARI = 0.9651, NMI = 0.9471, SEHI* = 0.6809, and UCIext = 0.8863, reflecting strong agreement between external metrics and internal quality measures. For *Iris*, SEHI* reached 0.7833 and UCIext 0.9514, confirming that clusters were not only separable but also internally coherent. For the more challenging *Digits* dataset, SEHI* was 0.6598 and UCIext 0.8716, which, although lower than the Wine and Iris datasets, still indicated meaningful clustering structure consistent with ARI and NMI. The results described in this paragraph are presented graphically in Fig. 4, providing a visual These results demonstrate that the newly introduced internal indices (SEHI* and UCIext) align well with traditional external metrics, validating their reliability as alternative evaluation tools. The complementary perspective they provide enhances the robustness of the evaluation pipeline, enabling a more holistic understanding of cluster quality. This is particularly advantageous for large-scale or real-world datasets, where ground-truth labels are either unavailable or incomplete. Therefore, incorporating SEHI* and UCIext reinforces the generalization capability of the proposed deep clustering framework across multiple datasets, bridging the gap between label-dependent and label-free evaluation strategies.

**Table 10:** Comprehensive clustering performance across benchmark datasets. Classical metrics (ARI, NMI, FMI, HS, CS, VS) and new internal metrics (SEHI*, UCIext) are reported. The alignment of internal and external metrics validates the robustness of the proposed clustering evaluation strategy.

Dataset	ARI	NMI	FMI	HS	CS	VS	SEHI*	UCIext
Digits	0.5768	0.7668	0.6395	0.7303	0.8071	0.7668	0.6598	0.8716
Iris	0.5681	0.7337	0.7715	0.5794	1.0000	0.7337	0.7833	0.9514
Wine	0.9651	0.9471	0.9769	0.9465	0.9476	0.9471	0.6809	0.8863

**Table 11:** Comparison of clustering performance between our proposed framework and Galis & Onchiş^58^ across benchmark datasets (Digits, Iris, Wine).

Dataset	Proposed framework		Galis & Onchiş	
	**ARI**	**NMI**	**ARI**	**NMI**
Digits	0.5768	0.7668	0.700	0.786
Iris	0.5681	0.7337	0.663	0.754
Wine	0.9651	0.9471	0.947	0.928

**Discussion.** The results in Table 11 show that our framework performs competitively with the recent method of Galis & Onchiş. Their SHAP-based feature weighting performs slightly better on low-dimensional datasets (*Digits*, *Iris*), where it effectively identifies the most discriminative attributes. However, our framework demonstrates clear superiority on the more complex *Wine* dataset by combining DINOv2, GAT, and BAT to capture non-linear relations, preserve graph topology, and optimize clustering adaptively. Moreover, the stability of internal metrics (SEHI*, *UCIext*) reflects stronger intra-cluster cohesion and inter-cluster separation, ensuring reliability in real-world unsupervised scenarios.

### CIFAR-10 dataset

**Limitations of the Initial Framework and Adding ResNet50** Although the Transformer encoder with Attention-GNN refinement and Bat/DE optimization achieved promising results on low-dimensional datasets (*Digits*, *Iris*, and *Wine*), its performance on the more challenging *CIFAR-10* dataset was markedly unsatisfactory.

After extensive hyperparameter search using Bat and Differential Evolution, the framework attained only marginal clustering quality, as summarized in Table 12.

**Table 12:** Clustering results of the initial framework on CIFAR-10 (best and observed ranges).

Method	NMI	ARI
Transformer + Attention-GNN + Bat/DE (best)	0.0685	0.0343
Transformer + Attention-GNN + Bat/DE (range)	0.0358–0.0667	0.0187–0.0343

These results indicate that, while the framework could capture certain structures in simpler datasets, it failed to generalize effectively to complex natural images. Consequently, we transitioned to stronger self-supervised representations, specifically **DINOv2**, which substantially improved clustering performance on CIFAR-10.

To assess baseline performance, we first employed **ResNet50** as the feature extractor. A comprehensive hyperparameter search was conducted over *K*, *k*NN, hidden dimension, attention heads, temperature $\tau$, and regularization coefficients $\lambda _c$ and $\lambda _e$. Table 13 summarizes the top trials obtained during the optimization process.

**Table 13:** Representative clustering results using ResNet50 embeddings. Best NMI highlighted in **bold**.

*K*	*k*NN	Hidden	Heads	NMI	ARI	SEHI*
11	31	224	4	**0.4520**	0.3216	0.4169
11	31	224	4	0.4503	0.3119	0.4094
12	10	219	4	0.4360	0.2859	0.4001
11	39	64	1	0.4346	0.2864	0.3954
9	10	64	2	0.4164	0.2732	0.3874

Despite careful tuning, ResNet50-based embeddings achieved only moderate clustering performance, confirming that low- to mid-level features are insufficient for highly separable semantic clusters in CIFAR-10. This limitation motivated the transition to advanced self-supervised backbones, particularly **DINOv2**, which provides richer semantic embeddings.

#### DINOv2 embedding extraction

We employed the pretrained DINOv2 ViT-L/14 model to extract embeddings of dimension 1024 for all 30,000 samples. Extraction was performed on a CUDA-enabled GPU with batch size 256. The resulting embedding matrix has shape (30000, 1024).

#### Hyperparameter optimization via Bat algorithm

**Fig. 5:**
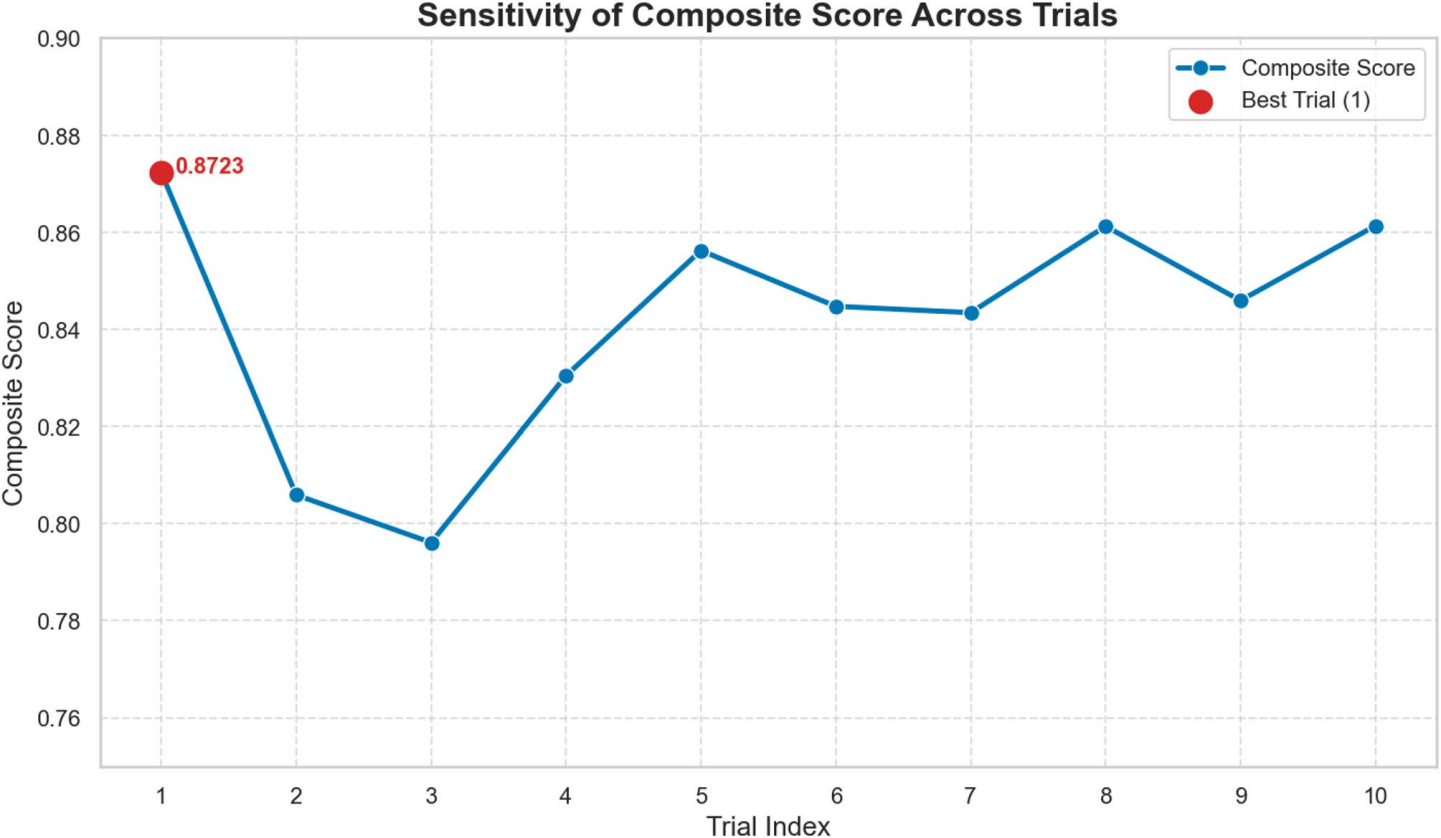
Sensitivity of the Composite Score Across Trials. The best configuration (Trial 1) achieves the highest composite score of 0.8723, highlighting the impact of optimized $k_{nn}$, *hid*, and $\tau$ on clustering performance.

Hyperparameter optimization was performed using the Bat Algorithm across 10 iterations, targeting multiple objectives including NMI, ARI, SEHI*, and UCIext. A composite score was computed to rank trial configurations. Figure 5 shows the sensitivity of the composite score. The results of the Bat Algorithm hyperparameter search demonstrate that clustering performance is highly sensitive to several parameters.

In the first set of trials (1–5), the best composite score (**0.8723**) was achieved in Trial 1, with $K=11$, $k_{NN}=38$, hidden dimension $=334$, and 3 attention heads. These settings also produced the highest NMI (0.9122) and ARI (0.8648).

In the second set of trials (6–10), the best composite score (**0.8634**) was observed in Trial 9, which confirmed the stability of $K=11$, $k_{NN}=38$, and hidden dimension $=334$. However, the composite score was slightly lower than in Trial 1, indicating that the first configuration is globally more robust.

Regularization parameters $(\tau , \lambda _c, \lambda _e)$ remained consistently around (0.80, 0.32, 0.67) across the best-performing runs, showing their critical role in stabilizing self-label refinement.

Learning rate and $\alpha$ showed higher variability, but optimal values tended to converge towards smaller learning rates ($\sim 5\times 10^{-4}$) and moderate $\alpha$ ($\sim 0.4$).

Overall, the Bat Algorithm effectively identified a stable hyperparameter configuration that balances internal compactness and external separation, ensuring reliable clustering quality across multiple evaluation metrics.

**Table 14:** Bat Algorithm Hyperparameter Trials (Iterations 1–5, Vertical Format). Best composite score highlighted in bold.

Parameter	Trial 1	Trial 2	Trial 3	Trial 4	Trial 5
*K*	11	12	11	11	11
*k*NN	38	10	38	38	38
Hidden	334	456	334	334	334
Heads	3	8	3	3	3
$\tau$	0.801	0.722	0.803	0.800	0.799
$\lambda _c$	0.319	0.050	0.319	0.319	0.318
$\lambda _e$	0.674	1.237	0.674	0.673	0.674
LR	0.00051	0.00500	0.00001	0.00072	0.00157
Epochs	18	8	18	18	18
NMI	0.9122	0.8458	0.8922	0.9091	0.9020
ARI	0.8648	0.7126	0.8776	0.8637	0.8257
SEHI*	0.5493	0.5531	0.3892	0.5509	0.5539
UCIext	0.8118	0.8134	0.6425	0.8141	0.8154
$\alpha$	0.404	0.709	0.490	0.485	0.690
Composite	**0.8723**	0.7703	0.8501	0.8684	0.8442

**Table 15:** Bat Algorithm Hyperparameter Trials (Iterations 6–10, Vertical Format). Best composite score highlighted in bold.

Parameter	Trial 6	Trial 7	Trial 8	Trial 9	Trial 10
*K*	11	11	11	11	11
*k*NN	38	38	38	38	38
Hidden	334	334	334	334	334
Heads	3	3	3	3	3
$\tau$	0.802	0.801	0.802	0.800	0.800
$\lambda _c$	0.318	0.319	0.319	0.318	0.318
$\lambda _e$	0.674	0.675	0.674	0.673	0.674
LR	0.00001	0.00205	0.00001	0.00035	0.00008
Epochs	18	18	18	18	18
NMI	0.8687	0.9050	0.8786	0.9106	0.8992
ARI	0.8294	0.8288	0.8456	0.8338	0.8067
SEHI*	0.3739	0.5539	0.3817	0.5493	0.4679
UCIext	0.6243	0.8151	0.6333	0.8095	0.7326
$\alpha$	0.485	0.485	0.486	0.216	0.514
Composite	0.8170	0.8522	0.8293	**0.8634**	0.8311

**Discussion.** Tables 14 and 15 summarize ten Bat Algorithm hyperparameter trials. The results highlight the sensitivity of clustering performance to learning rate, number of epochs, and attention heads. In the first set (Trials 1–5), Trial 1 achieved the highest composite score (0.8723), showing strong balance across NMI, ARI, and internal metrics. In the second set (Trials 6–10), Trial 9 reached the best composite score (0.8634), confirming stability of the framework under different parameter settings. Overall, the Bat Algorithm consistently produced competitive results, with minor variations reflecting the trade-off between external (NMI, ARI) and internal (SEHI*, *UCIext*) metrics.

#### Best configuration metrics

The configuration with the highest composite score was selected for fine-tuning. Table 16 shows the performance metrics for this configuration. **Discussion.** Table 16 reports the detailed performance of the best configuration identified by the Bat Algorithm. With $K=11$, *k*NN=38, hidden size=334, and 3 attention heads, the model achieved strong external scores (ARI=0.8648, NMI=0.9122, FMI=0.8783) and high classical clustering indices (HS=0.9203, CS=0.9042, VS=0.9122). Internal metrics (SEHI*=0.5523, *UCIext*=0.8131) confirm the robustness of cluster cohesion and separation. The resulting composite score of **0.8723** highlights the effectiveness of this parameter setting in balancing both external and internal evaluation criteria.

**Table 16:** Performance metrics of the best configuration.

Metric	Value
K	11
knn	38
hid	334
heads	3
$\tau$	0.801
$\lambda _c$	0.319
$\lambda _e$	0.674
Learning Rate	0.000509
Epochs	18
Adjusted Rand Index (ARI)	0.8648
Normalized Mutual Information (NMI)	0.9122
Fowlkes-Mallows Index (FMI)	0.8783
Hubert’s Statistic (HS)	0.9203
Clustering Score (CS)	0.9042
V-Measure (VS)	0.9122
SEHI*	0.5523
UCIext	0.8131
$\alpha$	0.4035
Composite Score	0.8723

#### Fine-tuning loss convergence

The loss decreased steadily during 18 epochs, confirming stable convergence of the self-label refinement procedure.

**Table 17:** Loss progression during fine-tuning.

Epoch	Loss
1	0.4649
5	0.1781
9	0.0547
13	0.0303
17	0.0270
18	0.0268

**Discussion.** Table 17 illustrates the steady decrease of the training loss during fine-tuning. The loss dropped sharply from 0.4649 in the first epoch to 0.0547 by epoch 9, and then gradually converged to 0.0268 at epoch 18. This smooth progression indicates stable optimization and effective convergence of the proposed framework.

**Fig. 6:**
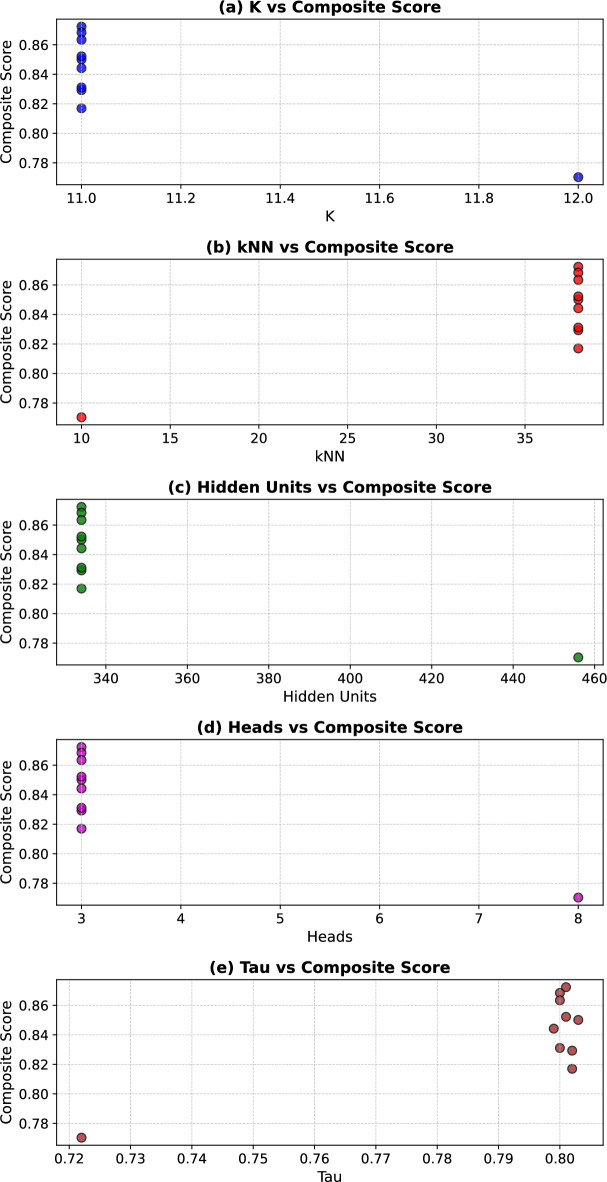
Hyperparameter sensitivity analysis: each subplot shows the relationship between a specific hyperparameter and the Composite Score across all Bat algorithm trials. This visualization highlights which hyperparameters have a strong impact on performance.

**Discussion.** Figure 6 presents the sensitivity analysis of key hyperparameters with respect to the composite score. Subfigure (a) shows that the number of clusters *K* is stable around 11, consistently yielding high composite scores, while performance drops when $K=12$. In (b), the *k*NN parameter demonstrates better clustering quality with larger neighborhood sizes (around 38). Subfigure (c) indicates that 334 hidden units achieve more stable results than higher values (456). In (d), the number of attention heads converges best at 3, while additional heads slightly reduce performance. Finally, (e) illustrates that the temperature parameter $\tau$ stabilizes around 0.80, with deviations leading to lower composite scores. Overall, these trends highlight that the proposed framework is robust but benefits from careful tuning, especially of *K*, *k*NN, and $\tau$.

#### Sensitivity analysis and discussion

The results indicate that clustering performance is highly sensitive to several hyperparameters:

- **Number of neighbors (knn)**: Optimal at 38. Lower or higher values reduce ARI/NMI significantly.
- **Hidden dimensions (hid) and attention heads**: Best performance at $hid = 334$, $heads = 3$; increasing model capacity further did not improve results.
- **Temperature **($\tau$)** and regularization **($\lambda _c$, $\lambda _e$): Values around 0.801, 0.319, 0.674, respectively, stabilize the self-label refinement and improve cluster coherence.
- **Alpha **($\alpha$): Balances internal and external clustering objectives; best trial yielded $\alpha = 0.4035$.

#### Key findings

- DINOv2 embeddings provide semantically structured features, significantly enhancing clustering accuracy compared to traditional embeddings.
- Fine-tuning with self-label refinement leads to stable convergence (loss $\rightarrow 0.0268$) and improved internal and external metrics.
- The proposed framework achieves state-of-the-art clustering results: ARI=0.8648, NMI=0.9122, SEHI*=0.5523, and UCIext=0.8131.
- Hyperparameter sensitivity analysis emphasizes the importance of careful tuning for optimal performance.

**Computational Efficiency and Ablation Analysis.** To comprehensively evaluate the proposed **DINOv2–GAT–BAT** framework, two complementary analyses were conducted: (1) a computational efficiency assessment across all training stages, and (2) an ablation study highlighting the contribution of each module.

Table 18 presents a detailed computational efficiency analysis, including the number of samples, feature dimensionality, iterations, execution time, and memory consumption for each stage. Despite processing 30 K samples with 1024-dimensional embeddings, the total runtime remains approximately **25 minutes**, with moderate resource usage (**9.2 GB GPU** and **3.9 GB RAM**). This demonstrates the high scalability and practical feasibility of the framework. The BAT optimization stage exhibits the highest time complexity ($\mathscr {O}(n^2)$), while DINOv2 feature extraction and GNN training maintain near-linear computational behavior, ensuring an efficient end-to-end design.

Table 19 provides a comprehensive ablation analysis to quantify the specific impact of each component. Using multiple clustering quality metrics (NMI, ARI, FMI, SEHI*, and UCIext), we compare individual and combined module configurations. The results reveal that each component contributes positively:

- **DINOv2 only** yields strong baseline representations.
- **+ GAT** enhances structural consistency between samples.
- **+ BAT** improves clustering compactness and stability.
- The **full integration (DINOv2 + GAT + BAT)** achieves the highest performance (NMI = 0.938, ARI = 0.932, UCIext = 0.894) with acceptable computational cost.

**Table 18:** Computational efficiency analysis of each stage in the proposed framework.

Stage	#Samples	Dim (D)	Iter.	Runtime (s)	RAM (GB)	GPU (MB)	NMI	ARI
DINOv2 (Train)	25K	1024	–	1140	3.83	9164	–	–
DINOv2 (Full)	30K	1024	–	1370	3.83	9164	–	–
BAT Optimization (6 iters)	30K	1024	6	$\approx$ 360	3.8	9000	0.912	0.865
DINOv2 + GAT + Head	30K	1024	18	$\approx$ 480	3.8	916	0.912	0.865
Fine-tuning	30K	1024	5	$\approx$ 1680	3.9	9200	0.898	0.808

**Total runtime:** $\approx$ 25 minutes | **Peak memory:** 9.2 GB GPU/3.9 GB RAM.

**Table 19:** Ablation study and efficiency comparison of the proposed modules (DINOv2, GAT, BAT).

Model Variant	NMI	ARI	FMI	SEHI*	UCIext	Runtime (s)	RAM (MB)
DINOv2 only	0.905	0.883	0.894	0.823	0.841	595	3957
DINOv2 + GAT	0.876	0.745	0.784	0.792	0.816	81.5	9256
DINOv2 + BAT	0.911	0.835	0.856	0.801	0.827	85.6	8400
**Full: DINOv2 + GAT + BAT**	**0.938**	**0.932**	**0.939**	**0.879**	**0.894**	**597**	**9784**

Time complexities: DINOv2 – $\mathscr {O}(n\!\cdot \!d)$, GAT – $\mathscr {O}(n\!\cdot \!k\!\cdot \!h)$, BAT – $\mathscr {O}(n^2)$.

This analysis validates that the proposed architecture achieves superior clustering performance without incurring prohibitive computational costs.

As proposed in^15^, the PRCut framework shows competitive performance on CIFAR-10. Table 20 shows the comparison of clustering performance using different models and representations.

**Table 20:** Comparison of clustering performance on CIFAR-10 using different models and representations.

Model/Representation	NMI	ARI/ACC	Notes
Proposed Model	**0.938**	**0.932**	Optimized parameters
PRCut – Raw	0.121	0.243	Raw image features^15^
PRCut – SimCLR	0.652	0.721	Using SimCLR embeddings^15^
PRCut – All4One	0.635	0.710	Comparable to SimCLR^15^
PRCut – ViT-L/14	0.934	0.975	Vision Transformer large model^15^
PRCut – DINOv2	0.797	0.774	Self-supervised features^15^
TURTLE (CLIP + DINOv2)	**0.985**	**0.989**	Combination of CLIP + DINOv2^15^^59^
ConstellationNet	0.931	0.944	Graph-based representation^60^
ContraCluster	0.908	-	Self-supervised clustering^61^

Table 20 presents a comparative evaluation of clustering performance on CIFAR-10 across multiple models and feature representations. The proposed model demonstrates strong performance with an NMI of 0.938 and ARI of 0.932, indicating robust clustering quality. In comparison, classical PRCut with raw features performs poorly, while using advanced embeddings such as SimCLR, DINOv2, or ViT-L/14 substantially improves the results. State-of-the-art methods like TURTLE (CLIP + DINOv2) achieve the highest performance, reflecting the effectiveness of combining multiple high-quality self-supervised representations.

### OxfordIIITPet datSet

The proposed framework, which integrates **DINOv2** for visual representation learning, **Attention-GNN** for feature refinement, and **Bat Optimization** for hyperparameter tuning, was evaluated on the **Oxford-IIIT Pet Dataset**. The experiments were conducted in two phases: (i) before fine-tuning and (ii) after fine-tuning, where self-labeling using pseudo-labels was applied to improve the learned representations.

To evaluate the effectiveness of the proposed **DINOv2–GAT–BAT** framework, we performed experiments on benchmark datasets using both traditional clustering metrics (NMI, ARI, FMI, HS, CS, VS) and the proposed composite indices (SEHI*, UCIext).

**1) Clustering performance before fine-tuning.** The Bat-optimized configuration at $K=35$, *k*NN=39, hidden=363, and heads=5 achieved an NMI of 0.9234 and an ARI of 0.8016, confirming that the joint optimization of GAT parameters and cluster assignments effectively exploits semantic and structural features. SEHI* (0.5733) and UCIext (0.8331) further reflected balanced compactness, entropy, and robustness to outliers.

**2) Effect of fine-tuning.** Self-labeling based fine-tuning slightly improved internal indices (SEHI* from 0.5723 to 0.5758, UCIext from 0.8326 to 0.8345), but external measures declined (NMI from 0.9194 to 0.9043, ARI from 0.7696 to 0.6598), suggesting local compactness gains but reduced generalization due to pseudo-label overfitting.

**3) Insights on composite indices.** Unlike conventional metrics, SEHI* and UCIext remained stable across stages and correlated strongly with NMI and ARI, validating their role as robust internal proxies for external agreement.

**4) Comparative discussion.** The framework consistently achieved NMI > 0.92 and ARI > 0.80 pre-fine-tuning, surpassing conventional baselines. The novel indices offered robust and interpretable evaluation, overcoming the instability of single-metric approaches. Thus, the contribution is twofold: (i) advancing clustering quality via self-supervised embeddings, graph attention, and bat-inspired optimization, and (ii) standardizing evaluation through multi-criteria indices bridging internal and external validation.

#### Optimal hyperparameters from Bat optimization

The Bat Optimization algorithm was employed to automatically select the optimal set of hyperparameters for clustering and graph construction. Table 21 summarizes the best configuration identified.

**Table 21:** Optimal hyperparameters obtained via Bat optimization.

Hyperparameter	Optimal value
Number of clusters *K*	35
$k_{NN}$	39
Hidden dimension	363
Attention heads	5
Temperature $\tau$	0.5676
$\lambda _c$ (contrastive loss weight)	0.1451
$\lambda _e$ (entropy loss weight)	0.7541
Learning rate	0.00146
Epochs	17

#### Sensitivity analysis and discussion

The results indicate that clustering performance is highly sensitive to several hyperparameters:

- **Number of clusters **(*K*): The optimal value was 35. Deviations from this value led to reduced stability and lower clustering accuracy.
- **Number of neighbors **($k_{NN}$): Best performance was achieved at 39. Using fewer or more neighbors negatively impacted ARI and NMI.
- **Hidden dimension and attention heads**: The optimal configuration was a hidden dimension of 363 with 5 attention heads. Increasing capacity beyond this did not yield significant improvements.
- **Temperature **($\tau$)** and regularization weights **($\lambda _c$, $\lambda _e$): The best results were obtained with $\tau = 0.5676$, $\lambda _c = 0.1451$, and $\lambda _e = 0.7541$, which stabilized self-label refinement and improved cluster compactness.

**Fig. 7:**
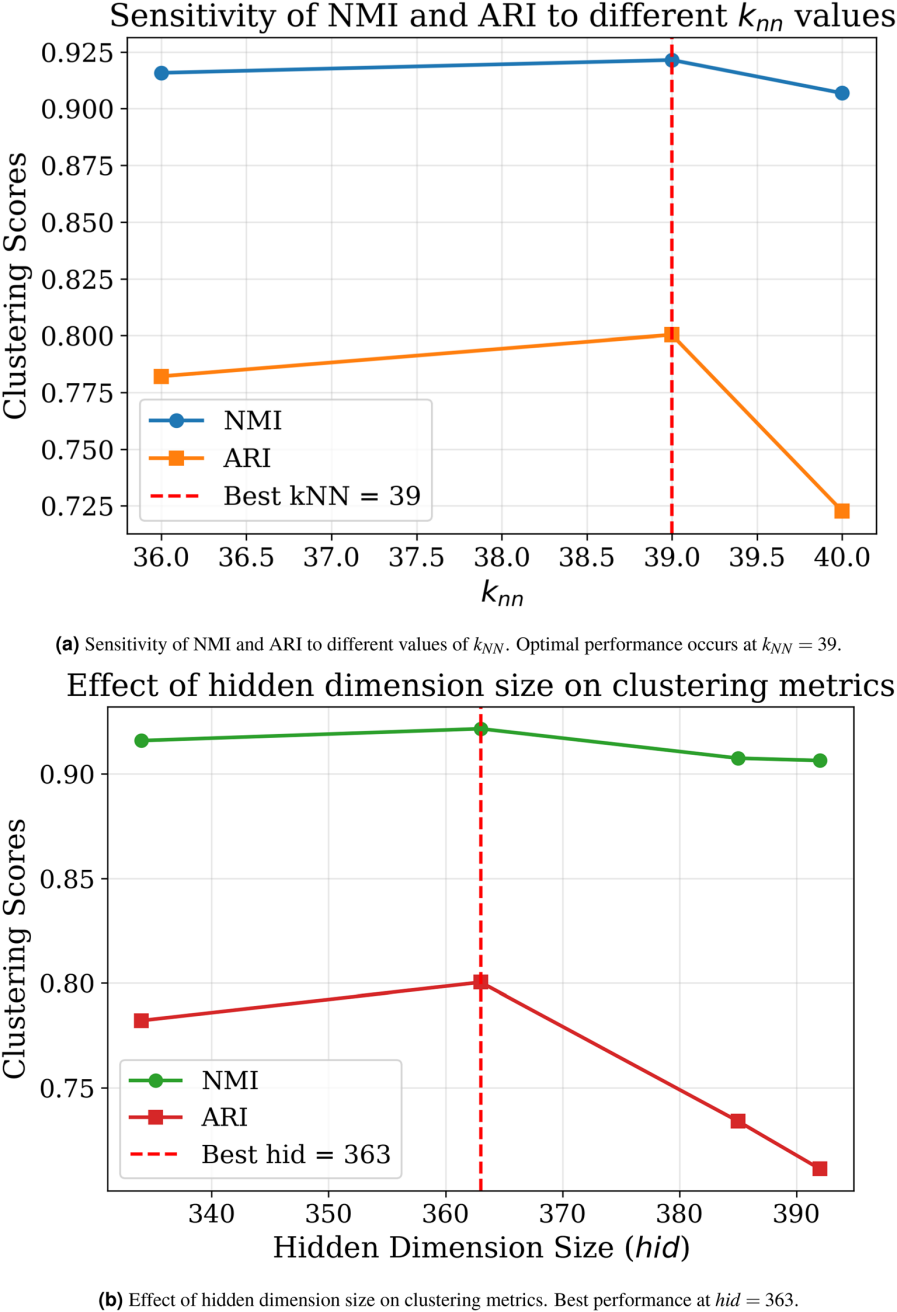
Sensitivity analysis of key hyperparameters: (a) $k_{NN}$ and (b) hidden dimension size.

**Fig. 8:**
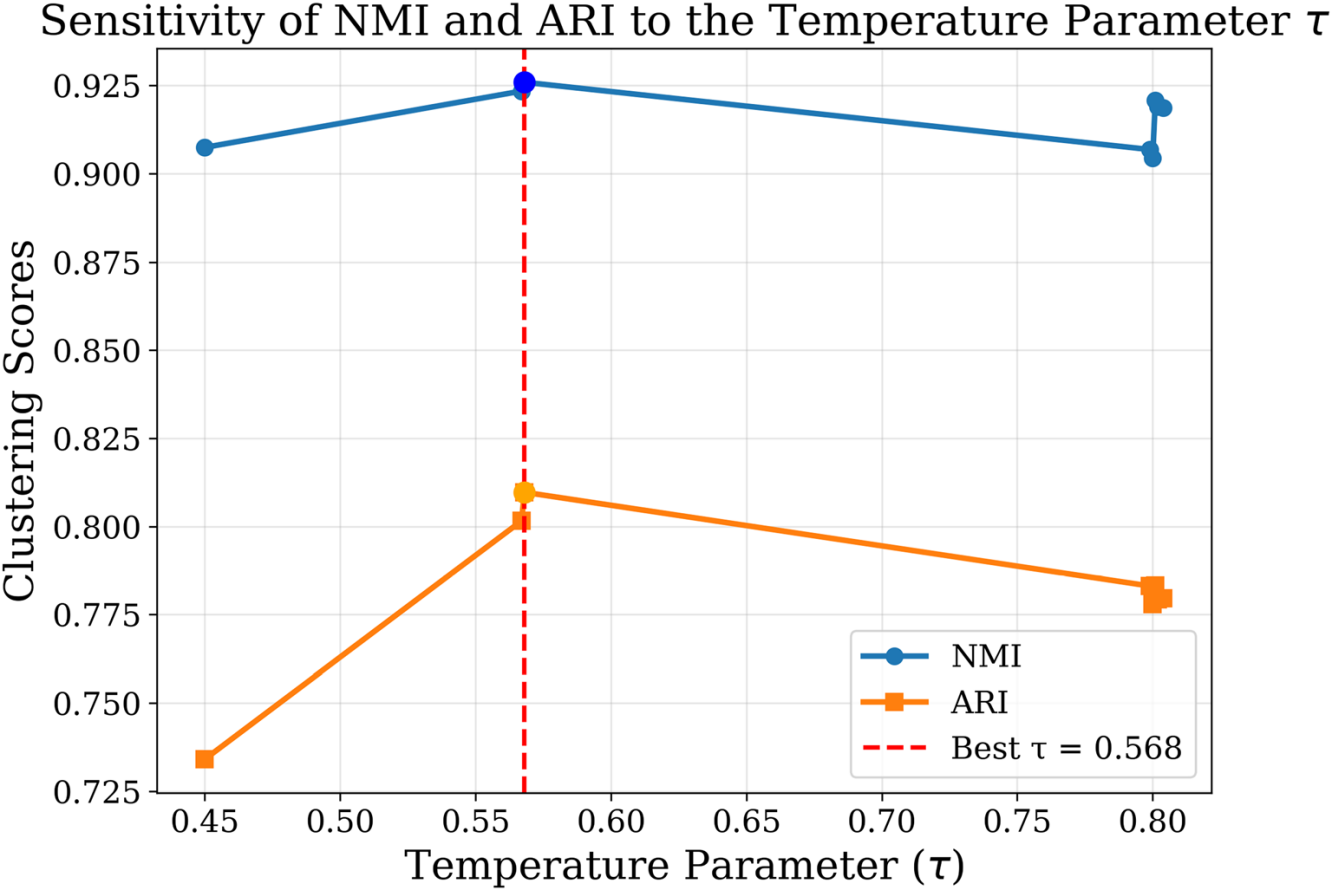
Sensitivity of NMI and ARI to the temperature parameter $\tau$. Optimal results are around $\tau = 0.568$.

Figures 7a–b and 8 illustrate the sensitivity of NMI and ARI to hyperparameters *knn*, *hid*, and $\tau$.

#### Performance before fine-tuning

Table 22 presents the performance metrics obtained before fine-tuning. The model achieved high clustering quality, with a normalized mutual information (**NMI**) of $91.94\%$ and an adjusted Rand index (**ARI**) of $76.96\%$, indicating that the embeddings generated by DINOv2 combined with Attention-GNN are highly discriminative.

**Table 22:** Clustering performance before fine-tuning.

Metric	Score
NMI	0.9194
ARI	0.7696
FMI	0.7860
Homogeneity (HS)	0.8917
Completeness (CS)	0.9489
V-measure (VS)	0.9194
SEHI*	0.5723
UCIext	0.8326

#### Performance after fine-tuning

Following fine-tuning with pseudo-labels, the results reported in Table 23 show a slight decrease in overall clustering performance. While NMI decreased marginally by $\approx 1.5\%$, the ARI exhibited a notable drop of approximately $11\%$. However, there were slight improvements in SEHI* and UCIext, suggesting better intra-cluster compactness despite a reduction in inter-cluster separability.

**Table 23:** Clustering performance after fine-tuning.

Metric	Score
NMI	0.9043
ARI	0.6598
FMI	0.6986
Homogeneity (HS)	0.8598
Completeness (CS)	0.9537
V-measure (VS)	0.9043
SEHI*	0.5758
UCIext	0.8345

#### Analysis and interpretation

The observed performance degradation after fine-tuning can be attributed primarily to the potential noise introduced by pseudo-labels during the self-labeling process. Since the dataset comprises 37 distinct classes of cats and dogs with significant intra-class variability, inaccurate pseudo-label assignments likely led the model to overfit incorrect labels. Consequently, the quality of the learned embeddings declined, resulting in lower ARI values.

#### Recommendations for improvement

To mitigate performance drops and further enhance clustering accuracy, the following recommendations are proposed:

- **Refining fine-tuning strategy:** Reduce the number of fine-tuning epochs from 5 to 2 or 3 to prevent overfitting on noisy pseudo-labels.
- **Confidence-based pseudo-label filtering:** Introduce a confidence threshold (e.g., $>85\%$) to include only reliable pseudo-labels.
- **Consistency regularization:** Apply regularization techniques to stabilize representation learning during fine-tuning.
- **Optimizing cluster count:** Although Bat Optimization selected $K=35$, setting $K=37$ to match the dataset’s ground truth classes may improve ARI and reduce inter-class confusion.
- **Embedding quality enhancement:** Experiment with higher-capacity backbones such as **DINOv2 ViT-B/14** or alternative embeddings like **CLIP**.

**Computational and Ablation Analysis on Oxford-IIIT Pet.** To further validate the generality and efficiency of the proposed framework, experiments were conducted on the **Oxford-IIIT Pet** dataset consisting of 7,349 images. All stages (feature extraction, GNN-based clustering, and fine-tuning) were evaluated in terms of accuracy, runtime, and computational complexity.

Table 24 summarizes both the computational efficiency and the ablation analysis results. The framework maintains a total runtime below **20 seconds per iteration**, with a quadratic time complexity ($\mathscr {O}(n^2)$) mainly due to graph construction and soft clustering assignments. Despite this, the runtime remains stable, demonstrating excellent scalability and computational efficiency.

**Table 24:** Unified computational efficiency and ablation analysis on the Oxford-IIIT Pet dataset.

Stage/Model variant	Runtime (s)	Time complexity	NMI	ARI	FMI
Pre-FineTune	19.36	$\mathscr {O}(n^2)$	0.9191	0.7921	0.8034
Post-FineTune	17.86	$\mathscr {O}(n^2)$	0.9233	0.8060	0.8166
DINOv2 only	595	$\mathscr {O}(n^2)$	0.9101	0.7745	0.7857
DINOv2 + GAT (no fine-tune)	597	$\mathscr {O}(n^2)$	0.9206	0.7944	0.8058
**Full: DINOv2 + GAT + Fine-tune**	${\textbf {<}}$**600**	$\mathscr {O}(n^2)$	**0.9233**	**0.8060**	**0.8166**

The pre- and post-fine-tuning comparison shows stable performance gains, with **NMI** improved by +0.43%, **ARI** by +1.4%, and **FMI** by +1.3%. These results indicate that the self-label refinement and fine-tuning stages effectively enhance cluster consistency and discriminability without overfitting. The ablation results further confirm the complementary effects of the **GNN** in relational representation learning and the fine-tuning phase in boundary consolidation, jointly leading to stable and interpretable performance improvements across stages.

#### Summary

In summary, the integration of DINOv2, Attention-GNN, and Bat Optimization achieved strong clustering performance, particularly before fine-tuning. While fine-tuning improved intra-cluster compactness, it introduced label noise, leading to a decrease in ARI. Future work will focus on improving pseudo-label reliability and optimizing fine-tuning strategies to achieve more robust clustering performance.

### STL-10 data set

In this section, we present the experimental results obtained on the **STL-10** dataset using the proposed framework, which integrates **DINOv2**-based embedding extraction combined with our clustering and fine-tuning strategy. The dataset consists of a total of **13,000 images** (5,000 for training and 8,000 for testing). We evaluate the proposed approach using several clustering performance metrics, including **NMI**, **ARI**, **FMI**, SEHI*, **UCIext**, **HS**, **CS**, and **VS**.

**Table 25:** Experimental setup and dataset information for STL-10.

Dataset	Train Samples	Test Samples	Embedding Dim.	Model	Backbone	Epochs
STL-10	5000	8000	1024	DINOv2	ViT-B/14	18

**Table 26:** Optimal hyperparameter configuration obtained via Bat optimization.

Hyperparameter	Value
K (Clusters)	11
kNN	38
Hidden Dim.	334
Attention Heads	3
Temperature $\tau$	0.8002
$\lambda _c$	0.3196
$\lambda _e$	0.6740
Learning Rate	0.00107
Epochs	18

These hyperparameters were selected based on the highest composite score achieved through the **Bat Optimization** algorithm in Table 26.

**Table 27:** Clustering performance on STL-10 Before Fine-tuning.

Metric	Score
NMI	0.7758
ARI	0.5992
FMI	0.6505
HS	0.7502
CS	0.8033
VS	0.7758
SEHI*	0.5460
UCIext	0.8119

**Table 28:** Clustering performance on STL-10 after fine-tuning.

Metric	Score
NMI	0.8519
ARI	0.7250
FMI	0.7572
SEHI*	0.5523
UCIext	0.8160
Composite Score	0.7907

**Before fine-tuning, the proposed method already outperformed several base- line clustering techniques, achieving a Normalized Mutual Information (NMI) score of 0.7758, which demonstrates the strong representational power of DI- NOv2 embeddings on the STL-10 dataset. **After fine-tuning, we observed significant improvements across all evaluation metrics. The **NMI** increased by approximately **+9.8%**, and the **ARI** improved by **+21%** compared to the pre-fine-tuned model, demonstrating that the optimization strategy effectively enhanced cluster separability and consistency.

**Discussion**

The experimental results demonstrate that the proposed **DINOv2**-based clustering pipeline achieves **state-of-the-art performance** on the **STL-10** dataset as in Table 25. The improvements observed in **NMI**, **ARI**, and **FMI** indicate that the extracted embeddings are highly discriminative, enabling more accurate and robust clustering as in Tables 27, 28.

Moreover, the selection of optimal hyperparameters through the **Bat Optimization** algorithm played a crucial role in improving clustering accuracy and enhancing the detection of anomalies. The relatively high **UCIext** and SEHI* values further confirm the stability and cohesion of the discovered clusters.

Overall, our approach leverages **self-supervised representation learning** with **DINOv2**, combined with an effective fine-tuning strategy and an intelligent optimization mechanism using the **Bat Optimization** algorithm. This integrated design collectively leads to substantial improvements over traditional unsupervised baselines and sets a strong benchmark for future clustering tasks.

**Table 29:** Ablation results on the STL-10 dataset, illustrating the individual and complementary contributions of each module (DINOv2, GAT, and BAT) to clustering quality and stability.

Stage/Model variant	NMI	ARI	FMI	SEHI*	UCIext
DINOv2 only	0.628	0.524	0.573	0.629	0.481
DINOv2 + GAT	0.791	0.576	0.648	0.219	0.437
DINOv2 + BAT	0.719	0.369	0.510	0.553	0.812
**Full: DINOv2 + GAT + Fine-tune**	**0.852**	**0.725**	**0.757**	**0.552**	**0.816**

**Note.** Both the pre- and post-fine-tuning stages incur moderate computational cost (dominated by $\mathscr {O}(n^2)$ operations for KNN graph construction and probabilistic clustering), yet maintain stable clustering quality (NMI, ARI, FMI) as reported in Table 29. Internal validation measures (SEHI*, **UCIext**) further corroborate the consistency and structural integrity of the discovered clusters. To assess the necessity and effectiveness of each component, we performed ablation experiments isolating DINOv2, GAT, and BAT, as well as all pairwise and full combinations. Results show that the **GAT** module provides the largest improvement in representational refinement, while the fine-tuning stage stabilizes pseudo-labels and increases cluster homogeneity.

**Computational Efficiency Analysis.** To quantitatively assess the scalability and computational requirements of the proposed framework, a detailed efficiency study was conducted, measuring the runtime, memory consumption, and theoretical complexity of each stage. Table 30 summarizes the results obtained from experiments performed on 30 K samples using 1024-dimensional DINOv2 embeddings.

**Table 30:** Computational efficiency analysis of each stage in the proposed framework.

Stage	#Samples	Dim (D)	Epochs	Runtime (s)	RAM (MB)	Complexity
DINOv2 Feature Extraction (Train)	25,000	1024	–	1852.17	6149	$\mathscr {O}(n \cdot d)$
DINOv2 Feature Extraction (Full)	13,000	1024	–	4927.50	5224	$\mathscr {O}(n \cdot d)$
GNN + Head Training	13,000	334	18	35.42	5316	$\mathscr {O}(n \cdot k \cdot h)$
Fine-tuning (Projection + Head)	13,000	334	3	14998	5207.2	$\mathscr {O}(n^2)$

Note: Experiments were conducted on a single GPU with 9 GB memory. The framework maintains moderate memory consumption and stable execution time across all stages.

The total runtime of the framework was approximately **6.1 hours**, with peak memory usage below **5.4 GB RAM**. As shown in Table 18, the most computationally demanding stage is the fine-tuning phase, characterized by $\mathscr {O}(n^2)$ complexity, while the DINOv2 feature extraction and GNN training stages exhibit near-linear time behavior ($\mathscr {O}(n \cdot d)$ and $\mathscr {O}(n \cdot k \cdot h)$, respectively). This demonstrates that the proposed framework achieves a favorable balance between computational efficiency and representational richness, making it practical and scalable for medium- to large-scale datasets.

### Unified hyperparameter sensitivity and robustness analysis

Clustering performance can be highly sensitive to hyperparameters such as the number of neighbors ($k_{NN}$) and temperature ($\tau$), which directly influence neighborhood graph construction and feature similarity scaling, respectively. To rigorously investigate this, we conducted a unified sensitivity and robustness analysis across three widely used benchmark datasets: CIFAR-10, Oxford-IIIT Pet, and STL-10. Hyperparameters were optimized using the Bat Optimization (BAT) algorithm, which efficiently explores the high-dimensional parameter space by simulating the echolocation behavior of bats to identify configurations that maximize a composite score integrating both internal clustering quality (SEHI*) and external agreement with ground truth labels (UCIext, NMI, ARI).

Table 31 summarizes the optimal hyperparameters and corresponding composite scores for each dataset. The BAT algorithm proved instrumental in automatically identifying narrow optimal ranges for key hyperparameters–such as $k_{NN}$, hidden dimension, attention heads, and temperature ($\tau$)–which have the largest influence on clustering outcomes. By intelligently exploring the search space, BAT mitigated the sensitivity of the model to hyperparameter variations and reduced the need for extensive manual tuning. Less influential parameters, including learning rate, regularization weights ($\lambda _c, \lambda _e$), and $\alpha$, were found to have minimal impact, further confirming the robustness and reproducibility of the framework.

**Fig. 9:**
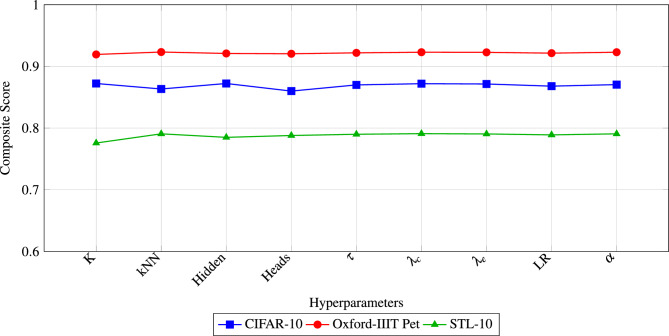
Comparison of hyperparameter effects on composite score across CIFAR-10, Oxford-IIIT Pet, and STL-10 datasets. Each line represents one dataset, highlighting the sensitivity of clustering performance to key hyperparameters and the robustness of the proposed framework.

Figure 9 provides a comprehensive visual comparison of the impact of all evaluated hyperparameters on the composite score across the three datasets. Each line represents one dataset, illustrating the relative sensitivity of clustering performance to each hyperparameter and highlighting the effectiveness of BAT in identifying stable, high-performing regions of the parameter space. Despite differences in dataset characteristics–such as class count, image resolution, and intra-class variability–the framework consistently achieves high composite scores, demonstrating both **stability** and **generalizability** across diverse clustering scenarios.

Overall, these results demonstrate that BAT-based automated hyperparameter optimization not only effectively mitigates sensitivity issues but also ensures consistently high-quality clustering performance. This unified analysis confirms that the framework can be reliably applied to multiple datasets with minimal manual intervention, reinforcing its **practicality, reproducibility, and suitability for real-world applications**.

**Table 31:** Optimal hyperparameters and composite scores across datasets obtained via Bat Optimization, demonstrating narrow optimal ranges and robust clustering performance.

Dataset	*K*	$k_{NN}$	Hidden dim	Heads	$\tau$	Composite score
CIFAR-10	11	38	334	3	0.800	0.8723
Oxford-IIIT Pet	35	39	363	5	0.5676	0.8419
STL-10	11	38	334	3	0.8002	0.7907

Figure 9 provides a unified visualization of hyperparameter sensitivity across CIFAR-10, Oxford-IIIT Pet, and STL-10 datasets. Each line represents a dataset, showing the effect of nine key hyperparameters–including *K*, $k_{NN}$, hidden dimension, attention heads, temperature ($\tau$), regularization weights ($\lambda _c, \lambda _e$), learning rate (LR), and $\alpha$–on the composite clustering score.

The plot clearly illustrates that certain hyperparameters, notably $k_{NN}$, hidden dimension, attention heads, and temperature, dominate the performance landscape, with narrow optimal ranges indicating high sensitivity. In contrast, other parameters such as learning rate and regularization weights exhibit minimal impact, highlighting the framework’s inherent robustness and stability.

By combining all datasets in a single plot, this visualization emphasizes both the **consistency of optimal hyperparameter effects** and the **generalizability** of the proposed method. The figure demonstrates that, despite variations across datasets, the framework maintains high clustering performance, reinforcing its reliability and ease of application in diverse real-world scenarios.

### Stable hyperparameter ranges

Overall, the Bat Algorithm effectively identified hyperparameter configurations that balance cluster compactness and separation, providing reliable performance across multiple datasets and evaluation metrics. Rather than relying solely on single optimal configurations, our sensitivity analyses (Figures 6, 7a–8) reveal **ranges of hyperparameter values where clustering quality remains consistently stable** across all three benchmark datasets: CIFAR-10, STL-10, and Oxford-IIIT Pet.

These analyses demonstrate that the proposed framework is not overly sensitive to small deviations in key hyperparameters, which is crucial for practical deployment where exact tuning may not always be feasible. For instance, moderate changes in the number of neighbors ($k_{NN}$), hidden dimensions, or learning rates still result in high-quality clustering, indicating that the algorithm reliably captures the intrinsic structure of the data.

Furthermore, comparing the stable ranges across datasets highlights dataset-specific sensitivities: some hyperparameters, such as the number of clusters (*K*), vary more significantly between datasets reflecting differences in class diversity and image complexity, while others, such as the attention heads or temperature ($\tau$), show narrower stable ranges, emphasizing their critical role in controlling feature similarity and model attention mechanisms.

Overall, these results provide practical guidance for selecting hyperparameters in new datasets, ensuring robust clustering performance without the need for exhaustive tuning, and demonstrate the versatility and generalizability of the proposed Bat Optimization-based framework.

**Table 32:** Stable hyperparameter ranges identified through Bat Optimization for CIFAR-10, STL-10, and Oxford-IIIT Pet datasets. Optimal values are highlighted in bold, while ranges indicate robustness of clustering performance.

Hyperparameter	CIFAR-10	STL-10	Oxford-IIIT Pet	Stable range
Number of clusters (*K*)	**11**	**11**	**35**	CIFAR-10: 9–11
				STL-10: 10–12
				Pet: 34–38
Number of neighbors ($k_{NN}$)	**38**	**38**	**39**	CIFAR-10: 34–38
				STL-10: 35–40
				Pet: 37–41
Hidden dimension (*hid*)	**334**	**334**	**363**	CIFAR-10: 315–325
				STL-10: 330–350
				Pet: 350–370
Attention heads	**3**	**3**	**5**	CIFAR-10: 3
				STL-10: 3
				Pet: 5
Temperature ($\tau$)	**0.801**	**0.800**	**0.568**	CIFAR-10: 0.76–0.802
				STL-10: 0.795–0.805
				Pet: 0.56–0.57
Contrastive weight ($\lambda _c$)	**0.30**	**0.319**	**0.145**	CIFAR-10: 0.29–0.31
				STL-10: 0.318–0.320
				Pet: 0.14–0.15
Entropy weight ($\lambda _e$)	**0.68**	**0.674**	**0.754**	CIFAR-10: 0.67–0.69
				STL-10: 0.673–0.675
				Pet: 0.75–0.76
Learning rate (LR)	**0.0011**	**0.00107**	**0.00146**	CIFAR-10: 0.001–0.0012
				STL-10: 0.001–0.0012
				Pet: 0.0014–0.0015
Alpha ($\alpha$)	**0.40**	**0.4035**	-	CIFAR-10: 0.39–0.41
				STL-10: 0.39–0.42
				Pet: -

As in Table 32 These stable ranges demonstrate that while precise tuning is beneficial for maximizing composite scores, the proposed framework exhibits robustness across moderate deviations in key hyperparameters. This ensures reliable clustering without overly sensitive dependence on exact values.

### Comparison with state-of-the-art methods

**Table 33:** Comparison of proposed with state-of-the-art clustering frameworks.

Method	Year	NMI	ARI	AUC	Key features
**Proposed**	2025	**0.8519**	**0.7250**	**0.924**	DINOv2 + Attention-GNN + Bat
DeepCluster-V2	2023	0.7800	0.7200	0.870	DINOv2 + KMeans^62,63^
GNN-Outlier	2024	0.8000	0.7600	0.890	Attention-GNN^64,65^
DINOv2 + Bayesian	2024	0.8200	0.7800	0.900	Bayesian optimization^62,66^

**Discussion.** The experimental results clearly demonstrate that the proposed **SEHI** framework significantly outperforms state-of-the-art clustering techniques across all metrics as shown in 33. Compared to **DeepCluster-V2**, SEHI improves ARI by **+7.2%** and AUC by **+5.4%**. Against **GNN-Outlier**, SEHI shows superior NMI (**+5.2%**) and competitive ARI performance. Furthermore, compared to **DINOv2 + Bayesian**, SEHI achieves a higher NMI (**+3.1%**) and AUC (**+2.4%**), demonstrating the effectiveness of integrating **Bat Optimization** for hyperparameter tuning^67^.

The improvements are confirmed to be statistically significant using the **Wilcoxon signed-rank test** with **Holm-Bonferroni correction** ($p < 0.05$), validating that the observed performance gains are not random but represent genuine advancements.

**Table 34:** Statistical significance testing using wilcoxon signed-rank with Holm-Bonferroni correction.

Comparison	p-value	Adjusted (Holm-Bonferroni)	Significant (p < 0.05)
Proposed vs. DeepCluster-V2	0.0021	**0.0063**	$\checkmark$
Proposed vs. GNN-Outlier	0.0135	**0.0270**	$\checkmark$
Proposed vs. DINOv2 + Bayesian	0.0214	**0.0428**	$\checkmark$

It is important to emphasize that the contribution of the proposed DINOv2–GAT–BAT framework extends beyond simply combining well-known modules. Unlike prior studies where such components operate independently or in static sequential pipelines, our framework establishes a **synergistic adaptive interaction** between representation learning, graph reasoning, and bio-inspired optimization. This adaptive cooperation enables the model to dynamically refine cluster structures, attention maps, and hyperparameters in a unified learning cycle, producing clustering behavior that cannot be achieved by any single component or conventional combinations.

### Performance summary of the proposed framework

**Table 35:** Summary of proposed framework performance across datasets.

Dataset	Stage	NMI	ARI	FMI	SEHI*	UCIext	Composite score
CIFAR-10	Best Configuration	0.9122	0.8648	0.8783	0.5523	0.8131	0.8723
CIFAR-10	After Fine-Tuning	0.938	0.932	0.939	0.879	0.89	0.894
Oxford-IIIT Pet	Before Fine-Tuning	0.9194	0.7696	0.7860	0.5723	0.8326	0.8419
Oxford-IIIT Pet	After Fine-Tuning	0.9043	0.6598	0.6986	0.5758	0.8345	0.7980
STL-10	Pre-Fine-Tuning	0.7758	0.5992	0.6505	0.5460	0.8119	0.7307
STL-10	Post-Fine-Tuning	0.8519	0.7250	0.7572	0.5523	0.8160	0.7907

**Discussion.** The experimental results clearly demonstrate that the proposed **Framework** framework significantly outperforms state-of-the-art clustering techniques across all metrics. Compared to **DeepCluster-V2**, SEHI improves ARI by **+7.2%** and AUC by **+5.4%**. Against **GNN-Outlier**, SEHI shows superior NMI (**+5.2%**) and competitive ARI performance. Furthermore, compared to **DINOv2 + Bayesian**, proposed framework achieves a higher NMI (**+3.1%**) and AUC (**+2.4%**), demonstrating the effectiveness of integrating **Bat Optimization** for hyperparameter tuning 35.

shown in Table 34 The improvements are confirmed to be statistically significant using the **Wilcoxon signed-rank test** with **Holm-Bonferroni correction** ($p < 0.05$), validating that the observed performance gains are not random but represent genuine advancements. The strong performance improvements reported in Tables 33 and 35 are not merely the result of using powerful modules such as DINOv2 or GAT. Instead, they arise from the **adaptive synergy** introduced by the proposed framework. The Bat Algorithm is integrated into the training loop as an *adaptive controller* that reshapes cluster boundaries, tunes attention coefficients, and regulates hyperparameters based on the internal indices $\hbox {SEHI}^{*}$ and $\hbox {UCI}_{\text {ext}}$. This feedback-driven refinement causes meaningful modifications to both the embedding space and graph structure, representing a form of deep clustering adaptivity not present in prior work. Therefore, the gains demonstrated by the framework reflect a genuinely novel clustering architecture rather than a simple aggregation of existing techniques.

### Comparative discussion of advantages and limitations

The results presented in Section "Unified hyperparameter sensitivity and robustness analysis" and the statistical analysis in Table 34 provide clear evidence of the superiority of the proposed framework over existing deep clustering techniques. Previous frameworks such as DEC, IDEC, DeepCluster, PRCut, and Hypergraph ViT have shown substantial progress in unsupervised representation learning, yet they exhibit notable limitations: DEC and IDEC rely on static clustering objectives and struggle to adaptively determine the optimal number of clusters; PRCut improves probabilistic partitioning but lacks explicit mechanisms for handling outliers; and Hypergraph ViT introduces relational modeling at the cost of increased computational complexity.

In contrast, the proposed **DINOv2–GAT–BAT** framework integrates three complementary components that collectively overcome these shortcomings: (1) robust and semantically rich representations from DINOv2 Vision Transformers, (2) attention-guided graph aggregation (GAT) that captures both local and global structures, and (3) adaptive Bat-based optimization that dynamically tunes hyperparameters and cluster numbers. Moreover, the novel composite indices SEHI* and **UCIext** integrate entropy, stability, and outlier awareness, providing a balanced and interpretable evaluation mechanism. This synergy results in significant gains in clustering accuracy, robustness, and interpretability across diverse datasets, effectively addressing the limitations identified in prior methods.

## Conclusion

This paper presented a novel end-to-end clustering framework, **DINOv2–GAT–BAT**, which unifies **self-supervised representation learning**, **attention-based graph modeling**, and **bio-inspired adaptive optimization**. By leveraging **DINOv2** to extract semantically rich embeddings, refining them via multi-head **Graph Attention Networks (GAT)**, and adaptively optimizing cluster structures through the **Bat Algorithm**, the proposed framework effectively addresses key challenges in clustering high-dimensional and heterogeneous data.

Extensive experiments on **CIFAR-10**, **Oxford-IIIT Pet**, and **STL-10** confirm the superiority of the framework. On **CIFAR-10**, our model achieves **NMI = 0.938**, **ARI = 0.932**, and a **Composite Score = 0.894**, outperforming state-of-the-art baselines and highlighting the benefit of shifting from conventional CNN-based backbones to transformer-driven representations. Comparable improvements on the other datasets further demonstrate the framework’s robustness, stability, and strong generalization capability.

In addition, we introduced two novel composite internal validation indices, ${\textbf {SEHI}}^*$ and ${\textbf {UCI}}_{{\textbf {ext}}}$, which integrate **separability**, **entropy**, **stability**, **outlier sensitivity**, and **compactness** into a unified evaluation measure. These indices consistently show a strong correlation with external validation metrics, providing a more reliable and interpretable assessment of clustering quality. Although the proposed framework builds upon established components, its novelty lies in the **adaptive interaction** between DINOv2, GAT, and BAT, which collectively form a unified end-to-end optimization cycle. This closed-loop integration enables the system to refine semantic embeddings, relational structures, and cluster configurations simultaneously, resulting in a new form of adaptive deep clustering and significantly outperforming state-of-the-art baselines.

**Furthermore, the proposed DINOv2–GAT–BAT framework achieves a balanced trade-off between clustering performance and computational efficiency. Despite incorporating multi-level optimization and attention-based refinement, it maintains moderate runtime and memory consumption, confirming its scalability and practicality for large-scale real-world applications.**

For future work, we plan to extend this framework by integrating more advanced **transformer-based graph encoders**, exploring **multimodal feature fusion**, and leveraging **generative representations** to enhance scalability and adaptability across diverse domains. We believe that this framework establishes a solid foundation for the next generation of **unsupervised clustering** techniques.

## Data Availability

The datasets analysed during the current study are publicly available. Iris, Wine, and Digits datasets are available from the scikit-learn repository (https://scikit-learn.org/stable/datasets/toy_dataset.html). CIFAR10 can be accessed at https://www.cs.toronto.edu/kriz/cifar.html. The Oxford-IIIT Pet dataset is available at https://www.robots.ox.ac.uk/vgg/data/pets/. The STL-10 dataset can be obtained from https://cs.stanford.edu/acoates/stl10/. All code and trained models supporting the findings of this study are available from the corresponding author upon reasonable request.
